# The Rise and Current Status of Polaritonic Photochemistry and Photophysics

**DOI:** 10.1021/acs.chemrev.2c00895

**Published:** 2023-09-08

**Authors:** Rahul Bhuyan, Jürgen Mony, Oleg Kotov, Gabriel W. Castellanos, Jaime Gómez Rivas, Timur O. Shegai, Karl Börjesson

**Affiliations:** † Department of Chemistry and Molecular Biology, University of Gothenburg, 412 96 Göteborg, Sweden; ‡ Department of Physics, Chalmers University of Technology, 412 96 Göteborg, Sweden; § Department of Applied Physics and Science Education, Eindhoven Hendrik Casimir Institute and Institute for Complex Molecular Systems, Eindhoven University of Technology, 5612 AE Eindhoven, The Netherlands

## Abstract

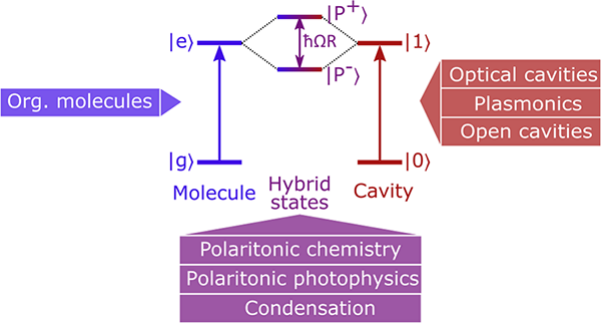

The interaction between molecular electronic transitions and electromagnetic fields can be enlarged to the point where distinct hybrid light–matter states, polaritons, emerge. The photonic contribution to these states results in increased complexity as well as an opening to modify the photophysics and photochemistry beyond what normally can be seen in organic molecules. It is today evident that polaritons offer opportunities for molecular photochemistry and photophysics, which has caused an ever-rising interest in the field. Focusing on the experimental landmarks, this review takes its reader from the advent of the field of polaritonic chemistry, over the split into polariton chemistry and photochemistry, to present day status within polaritonic photochemistry and photophysics. To introduce the field, the review starts with a general description of light–matter interactions, how to enhance these, and what characterizes the coupling strength. Then the photochemistry and photophysics of strongly coupled systems using Fabry–Perot and plasmonic cavities are described. This is followed by a description of room-temperature Bose–Einstein condensation/polariton lasing in polaritonic systems. The review ends with a discussion on the benefits, limitations, and future developments of strong exciton–photon coupling using organic molecules.

## 1 Introduction

Strong exciton–photon coupling is an extreme case of light–matter interactions. In everyday life, in the absorption event between a dye and a photon, the two entities can be described independently. In contrast, when their interaction becomes strong enough, hybrid light–matter quasiparticles, called polaritons, emerge. These are neither fully light nor matter, making their properties unique. The enhanced interaction between light and matter can be achieved by either an optical cavity, confining the electric field of a photon, or the enhanced field of surface modes or resonant (plasmonic) nanoparticles. The first theoretical model of strong exciton–photon coupling was introduced by Jaynes and Cummings in 1963^1^ and for the coupling to a surface plasmon by Agranovich and Malshukov in 1974.^2^ The first experimental reports were provided by Yakovlev et al. in 1975,^3^ and for the coupling to a surface plasmon by Pockrand et al. in 1982.^4^ The early works focused on the coupling of Rydberg atoms,^5−8^ and inorganic quantum wells,^9,10^ relying on low temperatures and optical cavities with a high quality factor.

The main focus of this Review is on strong exciton–photon coupling using organic molecules and its consequences on the excited state dynamics. The first experimental report on strong coupling using organic molecules was in 1998 by Lidzey et al.,^11,12^ one year after the theoretical description of Agranovich et al.^13^ A benefit of using organic dyes is their high transition dipole moments, resulting in high coupling strengths, which allows the use of low quality factor cavities that are easy to fabricate.^14^ Furthermore, the high exciton binding energy of organic molecules made it possible to reach low threshold polariton lasing and Bose–Einstein condensates at room temperature.^15−18^ In 2011, Schwartz et al. reported the photochemical process of a photoswitch within the strong coupling regime.^19^ This event was the starting point for the development of polaritonic photochemistry. The conceptual idea here is that an organic molecule is not a mere two level system but can perform many different photophysical and photochemical transformations that will be affected by the creation of polaritons. Today, strong coupling has been expanded over many fields of photophysics and photochemistry, which will be covered in great detail in later parts of this review.

Strong coupling is not restricted to electronic transitions; vibrational transitions can also be coupled to the electric field. The first coupled systems to a molecular vibration were realized in 2015.^20,21^ Soon thereafter, it was shown that vibrational strong coupling can affect the rate of chemical reactions.^22^ Since then, the field of strong coupling using organic molecules has been split into polaritonic photochemistry (coupling electronic transitions/electronic excited state) and polaritonic chemistry (coupling vibrational transitions/electronic ground state). Vibrational strong coupling has shown a rapid increase in research interest,^23−46^ and the reader is referred to other reviews including ones in this special issue for more information on the subject.^47,48^

This Review is organized as follows. We start with a short description of the fundamentals of light–matter interactions. This description recapitulates the essentials for the reader and introduces the nomenclature used by us. Then, different types of cavities are introduced. These cavities are used to increase the local electromagnetic field that organic molecules in close proximity experiences. How the interaction between the local electromagnetic field and the transition dipole moment of a molecular species can be expressed and experimentally analyzed is explained next. After this introduction of the theoretical foundations, the experimental landmarks within this field are described. This forms the main part of this review and is organized by the method by which the electric field is enhanced. First, planar cavities are discussed, and after that, plasmonic nanoparticles and nanoparticle arrays are presented. Furthermore, polariton lasing and Bose–Einstein condensation of organic exciton-polaritons are described in a separate section. The review ends with a summary that contains a personal reflection on possible future developments and directions within this exciting field.

## 2 Strong Light–Matter Interaction Essentials

The purpose of this section is to give the reader a conceptual understanding of strong light–matter interactions and a practical toolbox for analyzing strongly coupled systems. To start, we will give a recap of the basic concepts of light–matter interactions. We will further describe how the electric field can be enhanced to the strengths necessary to reach the strong coupling regime. This section allows the reader to track the physics and nomenclature used in later sections back to the fundamentals. For a more in-depth theoretical treatment of the subject, the interested reader is referred to previous reviews,^49,50^ and other reviews in this special issue.^51^

### 2.1 Basic Light–Matter Interactions

**Figure 1:**
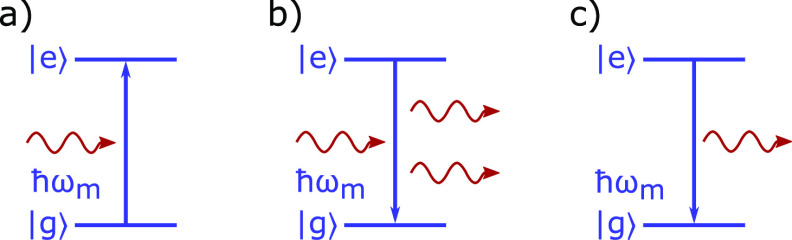
Sketch of the three different interactions between a photon and a two level system. (a) Absorption, (b) stimulated emission, and (c) spontaneous emission.

For the interaction between light and matter, Einstein postulated three fundamental processes between a photon and a two level system (Figure 1).^52^ During a photon absorption event, the two level system goes from the ground to the excited state, while the photon disappears. In the reverse process of emission, the two level system relaxes down to the ground state while simultaneously emitting a photon. This process can either be stimulated by another photon or be spontaneous. The energy of the photon interacting with the two level system needs to fulfill the Bohr frequency condition,^53^ which states that the energy difference between the two energy levels needs to be equal to the energy of the photon. A photon carries a discrete energy,^54^ which can be calculated by its frequency ν multiplied by Planck’s constant *h*. An alternative way to express this energy is by the reduced Planck’s constant  and the angular frequency of the photon ω = 2*πν*:
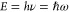
1

Transferring this two level picture is transferred to an organic molecule; those transitions occur between the electronic states. In order to make a transition from the ground state to the excited state, the molecule needs to absorb a photon. During this process, the electromagnetic field of the oscillating wave interacts with the electrons and cores of the atoms, forming the molecule. Quantum mechanically, this interaction can be treated as a time-dependent perturbation. In such a treatment, the energies of the ground and excited states are conserved, while the probability of finding the system in a certain state with respect to time can be derived from time-dependent perturbation. From the derivative of this probability, the strength of the interaction between the molecule and the electromagnetic field can be expressed as the transition dipole moment :^55^
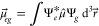
2where Ψ_*e*_ and Ψ_*g*_ are the eigenfunctions of the excited and ground states, respectively, and μ̂ is the electric dipole moment operator. The larger the transition dipole moment of the molecule, the stronger its interaction with an electromagnetic field and thus the higher the rate of absorption.

### 2.2 Interaction Enhancement Using Optical Cavities

In contrast to the previously described light–matter interactions, where the electromagnetic field and the molecule can be treated as separate entities, strong light–matter interactions result in a hybridization of both. In order to reach this strong exciton–photon coupling regime, the rate of exchange of energy between the exciton and the electromagnetic field needs to exceed the rate of energy dissipation in each system. This criterion is only met when molecules having large transition dipole moments interact with a considerable electromagnetic field.

**Figure 2:**
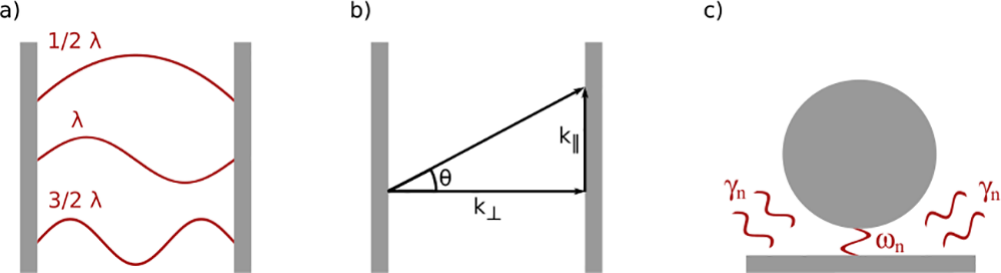
(a) Structure of a Fabry–Perot cavity with cavity modes at an integer number of λ/2. (b) Sketch of the two different wavevectors which are the origin of the dispersive behavior of a Fabry–Perot cavity. (c) Sketch of a plasmonic resonator treated as an open lossy system with complex-valued eigenmodes , where the imaginary part, describing the leakage rate for the *n*th cavity mode, contains all losses of the system (molecular, plasmonic, radiative).

A first approach to enhance the electromagnetic field is an optical cavity. An example is the Fabry–Perot cavity consisting of two planar mirror separated by a distance.^56^ The mirrors are typically distanced by a solid state spacer, although examples of cavities filled with a liquid exist.^57^ In this cavity, constructive and destructive interference increases the electromagnetic field at specific energies, modes (Figure 2a), which forms when the optical path length is an integer number *m* of half the wavelength λ:
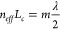
3where *n*_*eff*_ is the effective refractive index inside the cavity, and *L*_*c*_ is the geometric length of the cavity (the distance between the mirrors). The multiplication of *n*_*eff*_ and *L*_*c*_ results in an optical path length. To be able to fully describe strong exciton–photon coupling, the standing waves can be described by the quantized electromagnetic field. In the limit of zero photons inside the cavity, the quantum mechanical description predicts that a finite electromagnetic field is still present. This field is usually denoted as vacuum fluctuations and occurs due to Heisenberg’s uncertainty principle, appearing as virtual photons. So even in the absence of light, the molecules inside of a cavity can still interact with a virtual photon of the vacuum fluctuations. The strength of the vacuum electric field in an optical cavity can be determined by
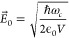
4where ω_*c*_ is the angular frequency of the cavity, ϵ_0_ is the vacuum permittivity, and *V* is the mode volume of the cavity.

Fabry–Perot cavities exhibit a dispersive behavior. The cavity mode energy *E*_*c*_ as a function of angle of incidence θ is given by the following expression (Figure 2b):^58^
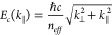
5where *c* is the speed of light in vacuum, *k*_⊥_ a wavevector, which is related to the length of the cavity *L*_*c*_ through
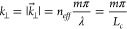
6and *k*_∥_ is the wavevector describing the in-plane momentum:
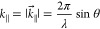
7which depends on the angle θ. Thus, for the case of incident light normal to the planar cavity, the in-plane momentum vanishes, and the cavity energy only depends on *k*_⊥_. On the other hand, *E*_*c*_ becomes larger when the angle of incidence increases.

From an experimental point of view, two different kinds of reflectors are typically used. One kind of mirror is metallic-clad mirrors, and the other is distributed Bragg reflectors (DBRs). Metallic mirrors are easy to fabricate and consist of only one mirror layer on each side of the spacer layer, whereas DBR are alternating layers of  optical length of two dielectrics with different refractive indices *n*_1_ and *n*_2_. The standing wave also has a larger penetration depth into DBRs compared with metallic mirrors, which increases the effective length of the cavity, resulting in a lower effective volume for molecules to be placed inside the cavity.

The quality factor of the cavity, *Q*, is a measure of how many optical cycles the wave makes before it decays by a factor *e*. The higher the quality factor, the higher is the number of rounds the photon takes within the cavity before leaving, which increases the interaction with the molecules. The quality factor can be experimentally determined through the energy and bandwidth of the cavity:
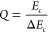
8

An advantage of DBR cavities is their much higher quality factor *Q* (on the order of 10^3^ – 10^6^), compared to metallic clad cavities (on the order of 10–100). However, high *Q* factors are often not needed to satisfy the criteria for entering the strong coupling regime when coupling to organic dyes.^14^ The thickness of metallic mirrors can therefore deliberately be kept low, in order to increase out- and in-coupling of light. Further, it has been shown that the refractive index mismatch between organic crystals and the surrounding in some cases is large enough to form a cavity with high enough *Q* to enter the strong coupling regime.^59−63^ This observation awakes exciting thoughts on strong coupling occurring in nature and on serendipitous strong coupling in, for instance, the research field of organic electronics, where length scales are of the right size to form modes in the visible part of the electromagnetic spectrum.

### 2.3 Strong Light–Matter Interactions

**Figure 3:**
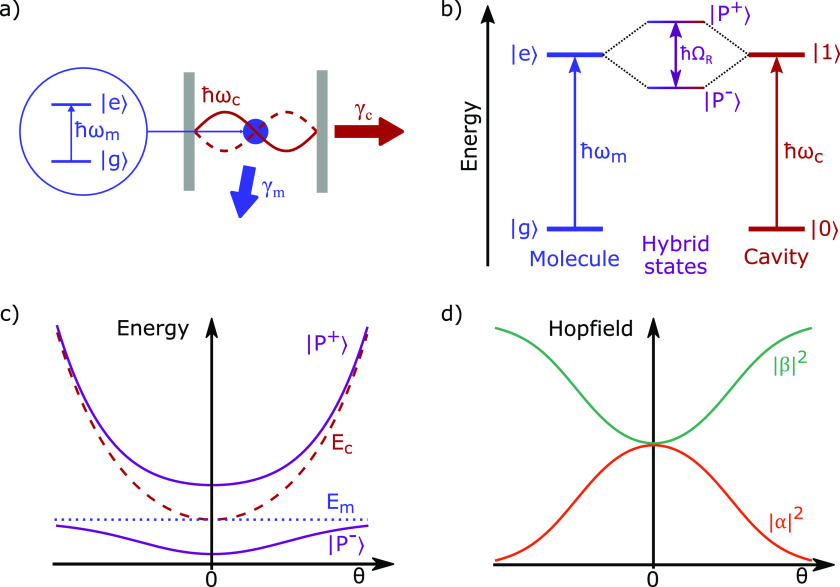
(a) Sketch of a two level system representing a molecule (blue) inside an optical cavity with the λ cavity mode (red) on resonance to a molecular transition. The dissipation from the system is indicated by γ_*c*_ for the cavity photon and γ_*m*_ for the molecule. (b) Energy diagram showing that the molecular transition (blue) and the resonance cavity mode (red) couple, leading to the formation of hybrid states, polaritons (purple), separated in energy by the Rabi splitting *ℏ*Ω_*R*_. (c) Dispersion of the formed polaritons (purple) with indicated behavior of the cavity mode (red) and exciton (blue). (d) Corresponding Hopfield coefficients |α|^2^ and |β|^2^ to the polariton dispersion in ‘c’.

In order to understand strong exciton–photon coupling to its full extent, a quantum description needs to be applied that includes both the molecular and light parts as well as their interaction. Within this context, the molecular part is reduced to a quantum two level system with a ground |*g*⟩ and an excited |*e*⟩ state. The energy difference between those two states is *ℏω*_*m*_ (Figure 3a,b). In order to introduce all possible transitions, three different operators are introduced. Namely the inversion operator , the raising operator , which brings the two level system from |*g*⟩ to |*e*⟩, and the lowering operator , which brings the two level system from |*e*⟩ to |*g*⟩. The light part in the model is described by quantized states, where an empty cavity is described by the Fock state |0⟩ and a single cavity photon is given by |1⟩. The creation operator , in general, increases the numbers of particles in a given state. In the given case, the Fock state is raised from |0⟩ to |1⟩ by creating a photon. In contrast, the annihilation operator â eliminates a photon, bringing the Fock state from |1⟩ to |0⟩. The energy of the photon is defined by *ℏω*_*c*_.

The last ingredient needed to define the coupled system is a description of the interaction between light and matter, where the operators of both parts are mixed together. The absorption process of a photon by a molecule is represented by the operator , where the photon is annihilated and the molecule raised to |*e*⟩. The reverse process of emission is represented by the operator combination of creating a photon and lowering the molecule to |*g*⟩, which is . Using the rotating wave approximation, the Jaynes-Cummings Hamiltonian is the sum of all three components:^1^
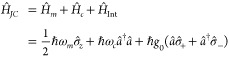
9where *g*_0_ is the magnitude of the coupling strength between the molecule and the photon.

The Jaynes-Cummings model describes only the interaction between a single molecule with a single cavity photon. However, the coupling strength *g*_0_ for a single molecule to the vacuum electric field is generally not large enough to realize strong exciton–photon coupling. In practice, there are many molecules placed in a single cavity in order to realize a high collective coupling strength. The model thus needs to be extended by *N* quantum two level systems, which interact with a single electromagnetic mode.^64^ For identical two level systems, collective spin operators ,  and  can be used^65^
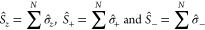
10and replacing the single molecule operators in the Jaynes-Cummings Hamiltonian, the Tavis-Cummings Hamiltonian is obtained^66,67^
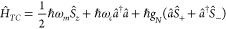
11

When the number of molecules *N* exceeds the number of excited states, the Holstein-Primakoff transformation can be applied.^68^ Now, the collective two level operators are transformed to bosonic operators b̂ and :
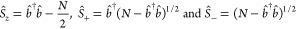
12

The Hamiltonian for the collective coupling for a large number of molecules then becomes
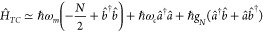
13where *g*_*N*_ is the magnitude of the coupling strength of many molecules collectively interacting with a single (virtual) photon.

The diagonalization of the Tavis-Cummings Hamiltonian leads to two bright new eigenstates, the upper polariton |*P*^+^⟩ and the lower polariton |*P*^–^⟩:^69^
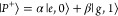
14a
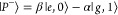
14b

The polaritons are linear combinations of an excited molecule in the absence of a cavity photon |*e*, 0⟩ and a ground state molecule in the presence of a cavity photon |*g*, 1⟩. The weights in the linear combination are α and β.

The interaction of *N* molecules with a single cavity photon results in two polaritons. The rest of the *N* – 1 states are fully excitonic, optically inactive dark states, which resemble the energy level of the molecular transition. They are often denoted as the exciton reservoir and have an tremendous influence on the excited state dynamics and thus on the photophysical and photochemical properties of the system, which are described in detail later in this review. When the exciton and cavity are on resonance, the energy splitting between the two polaritons is called the Rabi splitting, which magnitude can be expressed for the case of collective coupling in absence of dissipation as
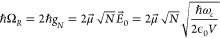
15

This equation shows the dependency of the coupling strength on the transition dipole moment of the molecules, μ⃗; the square root of the number of molecules, *N*, and the reciprocal mode volume, *V*. In other words, the square root dependence on the molecular concentration inside the cavity of the Rabi splitting is a way to prove that the molecule is in the strong coupling regime. This dependency also shows a disadvantage of optical cavities using DBRs. The high penetration depth of the field into the reflectors reduces the available volume for molecules, reducing the average molecular density and thus the coupling strength. Another aspect is that the transition dipole moment and the vacuum electric field are vectors, which underlines the importance of their relative orientation.^30,70−72^

The essential physics of the Tavis-Cummings Hamiltonian can be captured with a simplistic coupled harmonic oscillator model.^73^ Such a model is often used to describe macroscopic systems. For instance, two undamped harmonic oscillators, i.e., masses of *m* connected by a spring with a spring constant *k* to a wall. They oscillate with an angular frequency of ω. In order to describe their interaction, a third spring is introduced, connecting the two masses of the oscillators, leading to a periodic exchange of energy. As a consequence, the two oscillators cannot be described as independent entities anymore and the motion of the system can only be expressed in relation to the interaction spring constant *k*_3_:
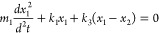
16a
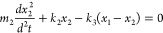
16bwhere the labels 1 and 2 refer to the two oscillators, and *x* is the displacement. Solving the differential equations, two new normal modes of the system are received:
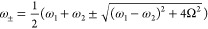
17where ω_±_ are the two new frequencies of the system, and Ω is the frequency splitting. A characteristic for strong coupling is the anticrossing behavior, which can be seen in eq 17. For the resonant case ω_1_ = ω_2_, the two new normal modes are separated by 2Ω, which depends on the interaction spring constant *k*_3_.

Using the coupled harmonic oscillator model to describe light–matter interactions, the cavity mode and the exciton are treated as harmonic oscillators coupled together by their interaction,
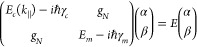
18where *E*_*c*_(*k*_∥_) and *E*_*m*_ are the cavity and molecular transition energies, respectively (Figure 3c), and *g*_*N*_ is the collective coupling strength. We also introduced here losses in the form of the cavity and molecule damping constants denoted as γ_*c*_ and γ_*m*_, respectively. The eigenvalues of the model give the energies of the lower *E*^–^ and upper *E*^+^ polaritons. The dependence of *k*_∥_ on the energies can be obtained by diagonalization of the Hamiltonian given in eq 18:
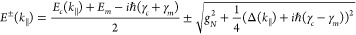
19where Δ(*k*_∥_) = *E*_*m*_ – *E*_*c*_(*k*_∥_) is the detuning, or energy difference between the molecular transition and the cavity mode. The Hopfield coefficients represent the photonic and excitonic contributions to the polaritons and they are given by
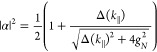
20
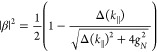
21

In the case of no detuning (Δ(*k*_∥_) = 0), the Hopfield coefficients to each polariton are both . The Rabi splitting is defined at resonance (zero detuning), where the energy separation between the polaritonic states is minimal (Figure 3d). Here, the coupling strength and Rabi splitting are related as
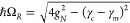
22

For the case of detuning, there are two possibilities. When the cavity mode energy is smaller than the molecular transition, the photonic contribution to the lower polariton increases (and vice versa for the upper polariton). This is often termed a red-detuned cavity. The opposite case is called blue-detuned, where the energy of the cavity mode is higher than the molecular transition, increasing the photonic contribution to the upper polariton. The coupled harmonic oscillator model is often used to determine the Hopfield coefficients and the Rabi splitting of strongly coupled systems from experimental data. The energies of the polaritons from a dispersion measurement are fitted by using this model, extracting the Hopfield coefficients and the Rabi splitting. A cause of warning should be made here; experimental dispersion data are recorded by varying the angle of incidence. eq 18 can be rewritten using an angle rather than k-space notation. However, fits should be performed in k-space. This is because the in-plane momentum is not conserved between the two polaritonic branches at a specific angle of incidence. After fitting has been performed, the results can be converted and displayed as a function of angle. Organic molecules show more than just a single transition, which is indicated by the two level system. In general, they have several vibronic transitions with different transition dipole moments. Therefore, it is sometimes necessary to extend the harmonic oscillator model with the amount of possible transitions *j*. The extended coupled oscillator model is also necessary for the analysis of systems with blended components, which all contribute to a strong coupling regime. In contrast to the simple 2 × 2 matrix, the extended version is tedious to solve analytically:
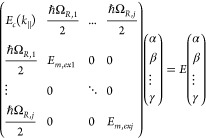
23

#### 2.3.1 Entering the Strong Coupling Regime

As already mentioned, a system is in the strong coupling regime if the rate of exchange of energy between the molecules and the cavity photon is faster than the dissipation rate of the cavity photon γ_*c*_ and the molecules γ_*m*_. Therefore, the coupling strength 2*g*_*N*_ needs to be larger than the mean value of the two dissipation losses:^74^
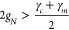
24

Experimentally, the determination of whether a system is in the strong coupling regime can be done from the transmission or reflection spectra. It can be observed from the Rabi splitting *ℏ*Ω_*R*_, which has a linear dependency on the coupling strength *g*_*N*_, which in turn has a square root dependency on the number of coupled molecules (see eq 15). A system can, for instance, be claimed to be in the strong coupling regime if the Rabi splitting *ℏ*Ω_*R*_ exceeds the full width at half-maximum (fwhm) of the cavity mode (*E*_*c*_) and the molecular absorption (*E*_*m*_):
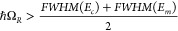
25

The exact point for the transition from the weak to the strong coupling regimes is however debatable.^75,76^ This transition should be seen as gradual^50^ because eq 19 can be analyzed in different ways. When γ_*c*_ = γ_*m*_, their contributions in eq 19 cancel each other, and it has been discussed that it is possible to express the strong coupling condition as a function of the difference between the rates of energy dissipation.^76^ Such definition is not advisable as it leads to nonphysical consequences in limiting cases. However, different ways of expressing the strong coupling condition in the form of the total rate of dissipation are possible, such as
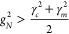
26

When the coupling strength *g*_*N*_ is further increased between light and matter, there are two more regimes that can emerge. For the so-called ultrastrong coupling regime (USC), the coupling strength reaches a significant fraction of the bare frequencies (0.1 ≤ *g*_*N*_/ω ≤ 1). Further, when the coupling strength exceeds the bare transition frequencies (*g*_*N*_/ω ≥ 1), the deep strong coupling (DSC) is reached.^77−80^ In the USC regime, the ground state is shifted in energy as well, acquiring a photonic contribution. This regime has gained in interest,^19,81−85^ and it has even been observed in cavity-free systems.^62^ The interested reader can learn more about USC and DSC in the reviews of Kockum et al.^79^ and Forn-Díaz et al.^80^

### 2.4 Interaction Enhancement Using Plasmonic Resonators

The above description of the light–matter coupling works well for low-loss, nonradiative, single-mode cavities, where one can unambiguously define the mode volume *V* and the number of coupled emitters *N* (as Fabry–Perot cavities typically are considered). In these cavities, the mirrors provide defined boundaries for both the electromagnetic field, as well as for the molecular volume, resulting in a collective coupling strength that scales with the molecular concentration,  (eq 15). In other words, there is a complete basis of discrete normal modes with real eigenfrequencies, and the effective mode volume is just related to the physical volume occupied by the mode. Small losses in such resonators are usually described by a perturbation of an ideal system, which broadens the eigenstates with a Lorentzian line shape, but leaves unchanged the resonance frequencies and field distributions.^86^ Although dielectric cavities (such as Fabry–Perot) allow high *Q*-factors to be achieved (up to ∼10^6^) their mode volume is basically limited by the diffraction limit (*V* ∼ λ^3^). This limit can be overcome by using another way of enhancing the electromagnetic field, namely by using a plasmonic resonator. Instead of relying on an optical standing wave, as in the Fabry–Perot cavity (diffraction limited), the oscillation of free charge carriers within a plasmonic resonator provides an enhancement of the electromagnetic field. The frequency and mode volume depend on the size and shape of the plasmonic resonator. Furthermore, arrays of resonators can be made where the resonant frequency of individual resonators is matched with the distance in-between resonators to form surface lattice resonances in which constructive interference and thus further enhancement of the electromagnetic field is achieved.^87^ Plasmonic resonators can confine light to very small mode volumes, on the order of *V* ∼ 10^–4^λ^3^, but this inevitably entails Ohmic losses and very low *Q* values (∼10). Such lossy systems have no true eigenmodes, but resonances with a given line width embedded in the continuum. However, to describe light–matter interactions at a quantum level, one must quantize the electromagnetic modes. This is much more difficult for open lossy systems compared to the well-defined Fabry–Perot cavity. Besides, as plasmonic resonators are open, comparable in size with the emitters themselves, and lossy, they are not well-defined in terms of *V* and *N*. The proportionality  is therefore questionable,^88^ although *ab initio* calculations show, that as an order of magnitude estimation, eq 15 may work well even for plasmonic resonators.^89^ Nevertheless, such lossy systems can no longer be described by real-valued eigenmodes but only with complex-valued frequencies , where the imaginary part gives the leakage rate (Figure 2c). The *Q*-factor is defined similarly to eq 8, as , which is consistent with an energy balance consideration. However, the imaginary part of the eigenmodes values gives a spatial divergence of the fields outside of the resonator and hence a divergence of the mode volume integral,^90^ which makes the definition of *V* quite challenging. For similar reasons, the common Purcell factor expression is not applicable for open lossy systems, as it is derived under the assumptions that normal (lossless) modes can be defined for the system, so that the local density of states is written as a sum of them, and this sum is dominated by a single mode.^91^

So plasmonic resonators are open non-Hermitian systems with complex-valued eigenmodes, and they cannot be reduced to a single-mode description within the Tavis-Cummings model. Instead, in general, one needs to work with a macroscopic QED Hamiltonian^92^ for multiple emitters, and have the plasmonic system described by the classical dyadic Green’s function . Such a Hamiltonian contains the frequency-dependent light–matter coupling expressed through the Green’s function as .^93^ However, the integral in this function ranges from zero to infinity over the entire continuum of frequencies, which makes it too computationally costly to solve.^94^

To avoid expensive numerical calculations, one needs to build a model system Hamiltonian represented in terms of discrete bosonic modes that describes the original system with enough accuracy. Nowadays, there are two main approaches for constructing an equivalent model Hamiltonian, which provide a few-mode quantized description of plasmonic resonators. One is based on finding the quasinormal modes (QNMs)^90,95^ of the resonator with complex-valued frequencies . Then, the quantized modes are defined as superpositions of the bosonic field operators with coefficients determined by the QNMs. These coefficients can be obtained from the poles calculations of the electromagnetic scattering matrix in the complex frequency plane.^96^ The resulting quantized modes are orthonormalized to obtain approximate discrete lossy modes. The negative sign of , which is demanded by the causality of electromagnetic fields, results in spatial divergence of the QNM fields outside of the resonator, thus making it difficult to properly define their normalization value. When using the QNMs method, one has to overcome this fundamental obstacle with the help of regularization techniques.^90,97,98^ More details about the QNMs approach can be found in refs (98−101).

An alternative approach^102−104^ does not require calculation and explicit quantization of the QNMs. Instead, it is based on fitting the full spectral density of a system obtained through classical electromagnetic simulations . A model system is constructed with a number of lossy and *interacting* modes that are linearly coupled to the quantum emitter and to an independent Markovian (spectrally flat) background bath. The Markov approximation allows to use the Lindblad master equation with a standard dissipation term. Furthermore, a Fano diagonalization results in a simple analytical solution for spectral density *J*_mod_(ω). Thus, it is not just a Lorentzian fitting widely used to quantize plasmonic systems in the quasistatic approximation. Here, the fitting procedure includes the intermode interaction, which allows reproducing the Fano-like resonances in the spectral density function. Fano-like resonances are typical for hybrid metallodielectric systems,^96^ and usually correspond to the interference of localized plasmons with standing-wave modes. So this fitting yields not only the frequencies and dissipation of the discrete modes, as well as all coupling constants between them and the emitters, but also the intermode interaction constants. All of this requires the dyadic Green’s function calculations as the only input. This approach is readily implementable and is a computationally efficient tool to construct a quantum description of light–matter interactions in plasmonic resonators. Note that both of the described approaches are also useful for modeling cavity-free polaritonic systems with self-hybridized modes.^61^

## 3 The Fabry–Perot Cavity

In the discussion of the Tavis-Cummings model (eq 13), we concluded that when *N* molecules couple to a single cavity mode, *N* + 1 new eigenstates form. Only two of the new states gain all oscillator strength from the incoming components. These are the polaritonic states, which are delocalized throughout the whole cavity and seen spectroscopically as a splitting of the excited state energy in the upper and lower polariton branches. The other *N* – 1 states are optically inactive and localized on the individual molecules. As discussed in the previous section, they have the same energy as the uncoupled exciton and are referred to as the exciton reservoir, exciton bath or as dark states.^105−108^ In early reports, the distinction between the exciton reservoir and uncoupled states was not always clear.^109,110^ The exciton reservoir is formed through the collective coupling of excitonic states to the cavity mode and by so doing “lend” their transition dipole moments to the polaritons. Uncoupled states on the other hand do not take part in the coupling to the cavity mode and therefore retain their transition dipole moment making them optically active. Uncoupled states are typically present at spatial locations within the cavity where the electric field is weak, such as at optical nodes.^111^ Furthermore, molecules with a transition dipole moment orthogonal to the electric field will be uncoupled because the coupling strength is proportional to the scalar product between the two components (eq 15). The commonality between the exciton reservoir and uncoupled states is their energetic distribution and their nonradiative interactions, such as their ability to perform photochemistry or relax nonradiatively. Much information on the energetic distribution and excited state lifetime of the exciton reservoir can thus be gained by observing the photophysics of the dye in the absence of a cavity.

**Figure 4:**
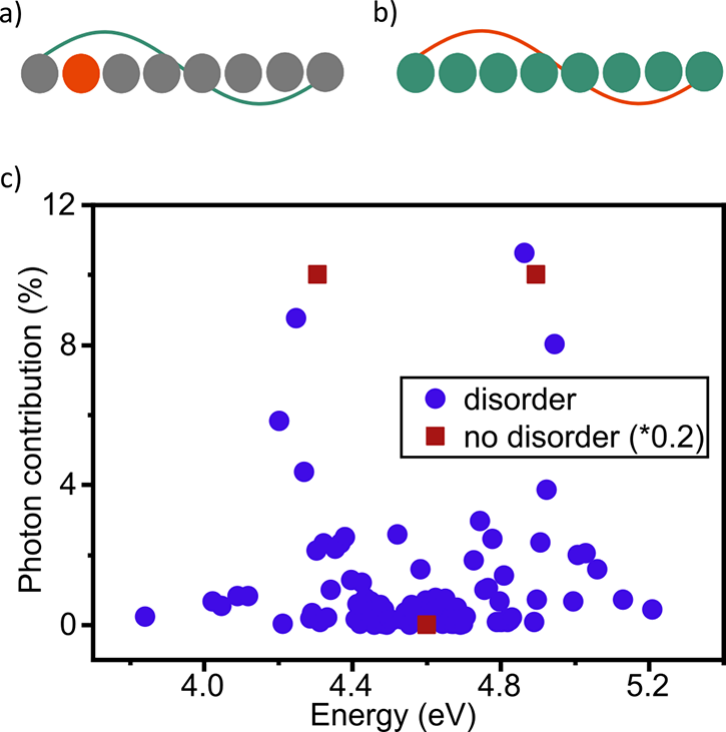
(a, b) Wave function overlap between a polariton and the exciton reservoir. The wave function of the polaritonic state is represented by a lambda wave, and the small spheres represent states in the exciton reservoir. Orange and green represent the initial and final excited states in an energy transfer reaction, respectively. The relaxation from a single exciton reservoir state to the polaritonic state is shown in ‘a’, and is a transition with a small wave function overlap. The relaxation from the polaritonic state to the exciton reservoir is shown in ‘b’ and is a transition where the small wave function overlap is compensated with the large number of available states in the exciton reservoir. (c) The effect of molecular transition broadening on the photonic contribution to the eigenstates. A system having a cavity mode coupled to 100 molecules with a narrow molecular transition (no disorder) and relatively broad molecular transition (disorder). In the narrow molecular transition coupling (red square), three distinct eigenstates are formed. The lower and upper polariton each having 50% photonic contribution along with 99 degenerate dark states having 0% photonic contribution and same energy as the molecular excited state. In the case of a realistically broad molecular transition (blue circles), the photonic contribution is no longer concentrated to two distinct states. Instead, the photonic contribution is with a varying degree distributed over many eigenstates. Reproduced from ref (117). Copyright 2021 John Wiley and Sons.

Although the exciton reservoir is not optically active, it hugely affects the photophysics of collective strong coupling.^110,112,113^ Polaritons are delocalized, whereas the exciton reservoir (and other molecular centered states) are localized.^114,115^ This results in a reduction of the exchange rate between the delocalized polaritonic states and molecular centered states (and vice versa) due to a wave function overlap mismatch (Figure 4a,b).^105^ The effect is similar to the Franck–Condon principle for vibronic transitions.^116^ The wave function mismatch scales with the number of molecules that collectively couple to each cavity mode, the delocalization number. In the ideal case of a joint collective coupling, the delocalization number approaches the total number of molecules in the cavity mode volume. Furthermore, the density of molecular centered states also scales with the total number of molecules. The result being that for transitions from polaritonic to molecular centered states, these two effects cancel per Fermi’s golden rule. However, for transitions from molecular centered to polaritonic states, the effective rate is reduced by a factor 1/*N*.

The view of collective strong coupling resulting in two polaritonic states and *N* – 1 dark states is an idealized view. Organic molecules generally exhibit spectrally broad transitions, and although very large relative coupling strengths are easily achieved, the coupling strength is often on the same sizescale as the exciton line width. To simulate the effect of the exciton line width on the photonic component of the generated hybrid states, Mony et al. simulated the coupling of 100 molecules to a single cavity mode.^117^ This simulation was done using the measured exciton line width and for the ideal case of a negligible exciton line width. Figure 4c shows this comparison, where for the ideal case (spectrally separated states) only two hybrid states contain a photonic component (50%) each, and the 99 remaining states are dark and energetically degenerate at the exciton energy. The two polaritonic states and the exciton reservoir are thus easily identified in such an ideal case. The picture changes when the experimentally measured exciton line width is used in the simulation. Now, the degeneracy is lifted, and a large distribution of energies is evident, although the density of states is highest at the exciton energy. Furthermore, there are now more than two states that contain a finite photonic contribution. Instead, a distribution of photonic contributions exists, where each polariton branch is marked by a couple of states having a slightly larger photonic contribution. Conceptually, similar results have also been seen by Groenhof et al.^118^ This puts a question mark on the effective number of dark states (effective N) in real-world systems and a need to develop experimental tools to determine this number. This determination should be done to determine if polariton photochemistry is dependent on the idealness of the polariton picture, where the molecule is treated as a perfect two-level system, or if the energy overlap between the polariton and exciton affects the photophysics of the strongly coupled system.

The exciton reservoir model has celebrated notorious triumphs, despite the above-mentioned concern of being, in many senses, too ideal. This success is based on a solid theoretical foundation and has been successfully applied to explain experimental observations. In the following sections, we will go through polariton photochemistry from an experimental point of view and often link back to how observations are compatible with the exciton reservoir model.

### 3.1 Polariton Relaxation Dynamics

**Figure 5:**
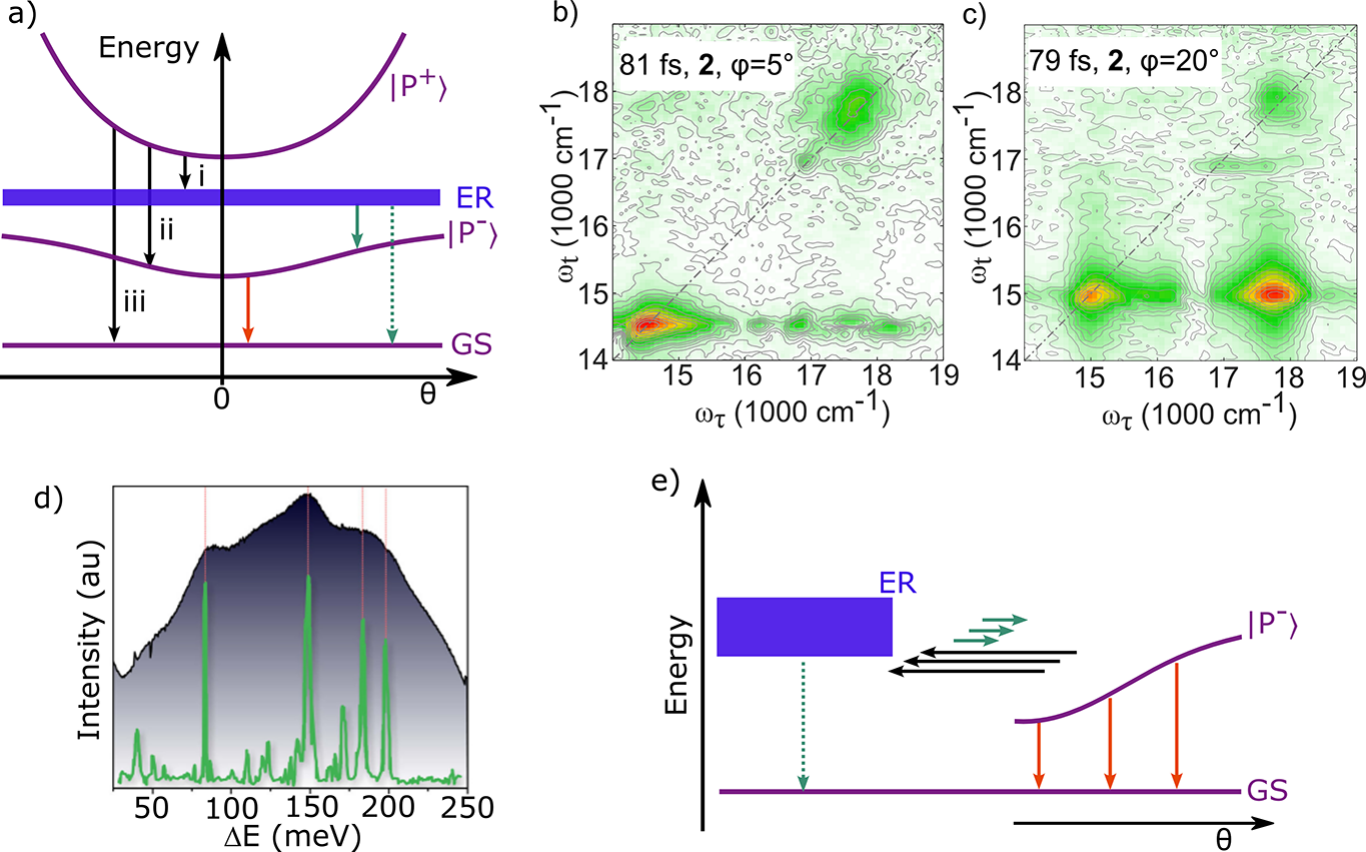
(a) Polariton relaxation pathways. From the upper polariton, the population can relax (black arrows) to the exciton reservoir (i), to the lower polariton (ii), or radiatively to the ground state (iii). From the exciton reservoir, the population can relax (green arrows) to the lower polariton or to the ground state. From the lower polariton, the population can relax radiativly (red arrow) to the ground state or transfer to the exciton reservoir. (b, c) Two-dimensional Fourier transform spectra of a TDBC J-aggregate in a red detuned cavity, monitored at (b) ϕ = 5° and (c) at ϕ = 20°. Here, broad-band visible light was used to probe the excitation dependence. Exothermic transitions can be followed by off-diagonal peaks in the lower right corner, and endothermic transitions can be followed by off-diagonal peaks in the top left corner. Reproduced from ref (132). Copyright 2020 Springer Nature. (d) Vibration assisted scattering. The shaded curve represents the total emission of the lower polariton (the spectrum was generated by summing all PL spectra at different detection angles) of a cavity containing TDBC. The total emission is plotted as a function of the energy separation, Δ*E*, from the exciton reservoir at 2.11 eV. The green spectrum represents Raman scattering. The peaks in the total emission from the lower polariton matches with the strongest Raman scattering peaks of the TDBC J-aggregate. Reproduced from ref (137). Copyright 2021 AIP Publishing. (e) The exciton reservoir can be viewed as being in a dynamic equilibrium with the lower polariton branch, which in turn can be approximated as a discrete set of states. This equilibrium heavily leans toward the exciton reservoir with the consequence being that the overall excited state lifetime often is dominated by the nonradiative rate of the exciton reservoir (green dotted arrow) rather than the polariton emission (red arrows).

The understanding of relaxation dynamics of polaritons is very important in order to use strong coupling phenomena in, for instance, polaritonic devices. Several femtosecond transient absorption spectroscopy (fs-TAS) studies have therefore been performed with this target in mind.^109,119−131^ Worth noting here is that it is not always easy to distinguish between decay of states and cavity induced artifacts. It is therefore good to be aware of possible pitfalls before performing pump–probe spectroscopy on optical cavities.^122^ Anyhow, after photoexcitation of the upper polariton, there are three relaxation channels possible (Figure 5a); (i) to the exciton reservoir,^110^ (ii) to the lower polariton,^132^ or (iii) directly to the ground state.^118^ The polariton can directly relax from the upper polariton at any *k*_∥_ to the exciton reservoir while transferring momentum and energy to the lattice. The rate of this transfer will be affected by the energy overlap between the two states. Both, the line width of the transition of the molecular dye and the coupling strength probably affect the rate of this relaxation. It should also be mentioned that it is experimentally demanding to probe the relaxation from the upper polariton to the exciton reservoir. This difficulty is due to the short lifetime of the upper polariton, on the order of a few tens of femtoseconds. Also the lifetime of the lower polariton is very short (sub ps).

A direct transfer from the upper to the lower polariton has only been confirmed in a few cases. Most likely because of the technological difficulties in distinguishing between direct transfer compared to a pathway via the exciton reservoir. To distinguish between these pathways, two-dimensional Fourier transform (2DFT) spectroscopy is required. This technique cross-correlation of the two states that participate in the energy transfer event. Figure 5b and c show such an experiment involving a J-aggregate,^132^ which is a popular type of molecule for this type of experiments.^133,134^ The excited state population, caused by direct excitation of the upper and lower polaritons, is along the diagonal axis. Relaxation between states is shown as off diagonal peaks, thus giving unambiguous experimental evidence for energy transfer events. At high *k*_∥_ and early times, a clear cross correlation peak is seen, indicating a direct relaxation from the upper to the lower polariton. At lower *k*_∥_ no such cross correlation peak is evident. This experiment suggests that the relaxation pathway depends on system parameters, such as *k*_∥_, which depends on cavity detuning and the viewing angle. The system under study was a J-aggregate, which has a very narrow absorption line width. The energy overlap between the exciton reservoir and the upper polariton is therefore very *k*_∥_ dependent. The observed effect can be phenomenologically explained by a difference in the relaxation rate from the upper polariton to the exciton reservoir. An interesting aspect of these experiments is the absence of clear cross correlation peaks above the diagonal axis. Peaks here would indicate energy transfer from the lower to the upper polariton and would be a sign of Rabi oscillations. A few other type of polaritons have also been examined by two-dimensional Fourier transform spectroscopy to try to understand the polariton relaxation dynamics.^135,136^

Emission, and thus a direct transition to the ground state from the upper polariton is not common but has been observed for J-aggregates.^110,138−141^ The observation has been explained from a thermal activation perspective.^138,139^ That emission from the upper polariton can occur as a consequence of a thermally activated repopulation of the upper polariton from the exciton reservoir. Latter, the theoretical aspects of upper polariton emission has been explored.^118^ It was then concluded that emission most likely happens at high *k*_∥_ due to the higher photonic nature of such polaritons.

#### 3.1.1 Exciton Reservoir to Lower Polariton Relaxation

From the exciton reservoir, relaxation can occur either to the lower polariton, to the ground state, or to any other molecular centered state such as a triplet state, exiplex, excimer, charge separated state etc. Relaxation from the exciton reservoir to the lower polariton can mainly be explained by two mechanisms, radiative pumping and vibrational assisted scattering.^112,118,137,142,143^ Radiative pumping is based on emission followed by an absorption event. The energy is localized on a single molecule in the exciton reservoir. This molecule relaxes from the Franck–Condon state to the geometry relaxed excited state. The resonance detuning between the cavity and the excited molecule changes as a result of this relaxation. Depending on the magnitude of the reorganization energy, it is actually questionable whether the molecule is still coupled to the cavity. From the geometry relaxed state of the molecule, it emits a photon, which then can be absorbed by the lower polariton. The efficiency/effectiveness of radiative pumping mainly depends upon the overlap integral between the excitonic emission and the lower polaritonic absorption.^142^ It therefore inversely varies with the energy difference between the relaxed excited state of the molecule and the lower polariton.

Vibrational assisted scattering is mainly observed in molecules having small reorganization energies (small Stokes shifts), such as J-aggregates.^112,137^ Coles et al. as well as Hulkko et al. observed that in TDBC J-aggregates, the cavity emission shows several local maxima (Figure 5d). The energy difference between these maxima and the exciton reservoir (note that the reorganization energy in TDBC J-aggregates is close to zero) matches the most pronounced Raman scattering peaks of the J-aggregate. The authors naturally linked the increased emission to the ability of the system to dissipate energy vibrationally. In a nonlinear organic dye, there are 3N-6 vibrational modes, where N is the number of atoms in the molecule. Vibrational assisted scattering has been shown only for Raman active modes and not for IR active modes. Given the large number of vibrations at different energies for an organic dye (typically 60–100), it is at present unclear why these vibrations do not form a continuous energy band that promotes relaxation through this channel.

Radiative pumping and vibrational assisted scattering are two competitive pathways for relaxation from the exciton reservoir to the lower polariton. Hulkko et al. explained how the Stokes shift (reorganization energy) of the molecule affects which of these pathways that dominates the relaxation.^137^ For molecules having large Stokes shifts (valid for most molecules), the overlap integral between the excitonic emission and the polaritonic absorption is large. Therefore, radiative pumping dominates over vibrational relaxation. J-aggregates have both a very small Stokes shift and a small emission line width. For TDBC, the emission is even close to overlapping the absorption. When such molecular systems are in the strong exciton–photon coupling regime, the lower polariton can be at significantly lower energies as compared to the molecular emission. The overlap integral is therefore close to zero, radiative pumping diminishes, and vibrational assisted scattering is the dominant pathway of relaxation from the exciton reservoir to the lower polariton. To summarize, radiative pumping is the most common relaxation mechanism in most organic dyes, whereas vibrational assisted scattering can become prominent in J-aggregates.

#### 3.1.2 Emission from the Lower Polariton

Generally, emission in strongly coupled systems is observed from the lower polariton branch, irrespective if excitation was made to the exciton reservoir or the upper/lower polariton.^14,144−149^ This is due to the fast decay from the upper polariton to the exciton reservoir in combination with the zero net transition dipole moment of the exciton reservoir, which results in no other possible emissive route than the lower polariton. Emission typically occurs strongest from *k*_∥_ = 0. However, in the lower polariton branch, the in-plane momenta can be changed only by a dynamic transfer back and forth to the exciton reservoir. Thus, this process has to occur in order to observe a difference in *k*_∥_ for the excitation and emission channels when exciting the lower polariton branch.^150^ Note, at the dye concentrations used in strong coupling experiments, intermolecular energy transfer is fast. The in-plane momentum thus is randomized in the exciton reservoir.

As the polariton has both light and matter components, the theoretical polaritonic lifetime should be limited by the component having the shortest lifetime. The magnitude of the lifetime of molecular excited states varies from a few hundreds of femtoseconds to nanoseconds. This is generally longer than the magnitude of the photon lifetime (often less than 50 fs), which then sets an upper boundary of the polaritonic lifetime. Several studies report polaritonic lifetimes on the order of the photon lifetime.^151−153^ However, a larger number of studies found polaritonic lifetimes on the order of or even longer than the molecular excited state lifetime.^109,111,126,149,154−156^ The variation of observed polariton emission lifetimes can be rationalized from the exciton reservoir theory as follows (Figure 5e): the exciton reservoir might be optically dark, but the value of the nonradiative rates to the ground state is inherited from the bare molecules. Furthermore, at the high molecular concentrations usually used in strong exciton–photon coupling experiments, the natural decay lifetime is dominated by the nonradiative rates. The exciton reservoir theory assumes that a rapid equilibrium (or at least the possibility of transfer in both directions) exists between the exciton reservoir and polaritonic states of different in-plane momentum (*k*_∥_). This equilibrium heavily leans toward the exciton reservoir, which then dominates the kinetics. In principle any observed lifetime of the lower polariton can be explained by this equilibrium, although the nonradiative decay rate of the molecule puts an upper boundary of the lifetime. It is however strongly nonintuitive that transfer from the lower polariton branch to the exciton reservoir is fast enough to out-compete polariton emission. Further, as the in-plane momentum is scrambled in the exciton reservoir, the polariton lifetime is angle independent.^149^ It is surprising that observed polariton lifetimes so often matches with the fluorescence lifetime in the weak coupling regime, although it could to some extent be explained by nonradiative rates being the dominant decay channel. An increase/decrease of radiative rates then only has a minor effect on the observed lifetime. Still, an open question is if this phenomenological explanation is enough or if other explanations are needed. For instance, it has been put forward that the lifetime of the lower polariton might be longer than expected from the cavity lifetime.^109^

The theory also explains why emission intensity is highest normal to the cavity.^148^ The photon contribution to the lower polariton is the highest, and the rate of transfer to the exciton reservoir is the lowest at low *k*_∥_. The population density thus have a tendency to redistribute toward low *k*_∥_ through the exciton reservoir. Such a redistribution can be simulated using rate-equations where the lower polariton branch is approximated by a discrete set of states (Figure 5e).^157^ Due to redistribution, it also sometimes happens that the emission from the lower polariton is a little bit blue-shifted as compared to polariton absorption. The proposed mechanism for this commonly observed phenomenon involves polariton to exciton reservoir energy transfer, followed by absorption of thermal energy and return to the lower polariton.^158^

In the ultrastrong coupling regime, the emission energy from the lower polariton becomes less angle dependent. Ultrastrongly coupled systems are therefore potential candidates for LED applications.^159,160^ Charged polaron polaritons are also of importance in this regards, and emission form such has been observed.^161,162^ Finally, polariton lasing has been observed from the bottom of the lower polaritonic branch.^15,18,163^ This will be discussed in detail in Section 5 of this Review.

### 3.2 Relaxation from Polaritonic to Molecular Centered States

**Figure 6:**
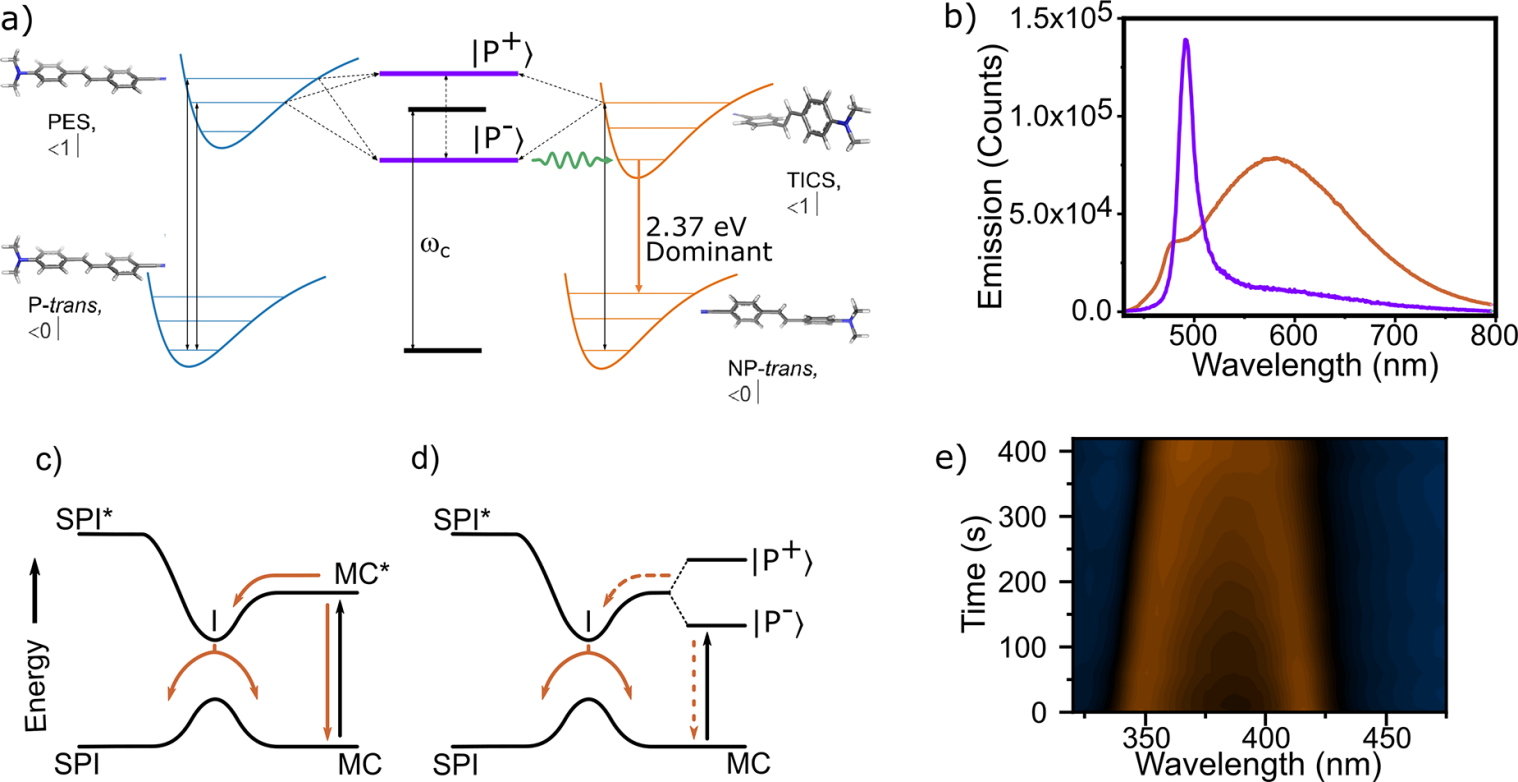
(a) In a molecule having the possibility to form a twisted intramolecular carge transfer state (TICS) on the excited state surface, the lower polariton can facility the energy funneling toward the TICS. Reproduced from ref (164). Copyright 2021 AAAS. (b) Emission from DABNA-2 in two differently tuned cavities. A large change in the ratio of polariton versus excimer emission is seen. Reproduced from ref (165). Copyright 2022 the American Chemical Society. (c) The energy landscape of the photoisomer couple spiropyran (SPI) and merocyanine (MC). After excitation of MC (black arrow), relaxation can occur (orange arrows) to both MC and SPI. (d) When MC is placed in the strong coupling regime, the excited state photophysical pathways are perturbed (dashed orange arrows). For instance, strong coupling can introduce an energy barrier from the lower polariton branch of merocyanine to the energy minima on the excited state surface (I), which affects the photoisomerization. Reproduced from ref (166). Copyright 2012 John Wiley and Sons. (e) Absorption as a function of irradiation time of norbornadiene in the strong coupling regime, showing a contraction of the Rabi splitting as norbonadiene is photoswitched into tetracycline. Reproduced from ref (117). Copyright 2021 John Wiley and Sons.

Organic molecules have a large number of degrees of freedom. This characteristic is because they are built out of covalent bonds, giving the molecule an opportunity for a large number of vibrations. They further respond to their environment, causing movement, a change in bonding strength, or even the breakage of bonds. Before starting to discuss transitions from polaritonic states to molecular centered states, it is important to mention the effect of inhomogeneous broadening of the exciton on the polariton line width. It has been shown that when the coupling strength is considerable larger than the exciton line width, the polariton line width approaches that of the homogeneous exciton line width (given that the cavity line width is not limiting).^151^ This indicates that two chemically different species (either two molecules being in different aggregational environments or two different molecules) can jointly couple to a single cavity mode as long as the coupling strength is sufficiently large. An example of such joint coupling was shown recently. The directional energy transfer between two rotamers having similar excitonic energy but different energy on the geometry relaxed excited state surfaces was studied (Figure 6a).^164^ As both rotamers have similar exciton energies, they collectively couple to the cavity mode, forming one upper and one lower polariton branch. The cavity was tuned so that the lower polariton branch was in energy located between the energy of the geometry relaxed excited states of the two rotamers. Then all energy was harvested toward the rotamer having the lowest relaxed excited state energy. The efficiency of this directional energy transfer improved as the energy gap between the lower polariton and the relaxed excited state decreased.

Another process on the excited state surface of an organic molecule is the formation of molecular trap states, such as excimers, which is a result of dimerization on the excited state surface. The interplay between such trap states and polaritons have been investigated.^165^ The result showed that whether one can observe polaritonic emission rather than excimer emission depended heavily on the tuning of the cavities (Figure 6b). For red-detuned cavities, which have a higher photonic contribution and lower energy of the lower polariton, the polaritonic emission was dominant. However, for blue-detuned cavities, the excimer emission was stronger. Similar conclusions were drawn for molecules exhibiting prompt and delayed excimer emission. For the case of prompt emission, it is not obvious if the observation can best be explained by kinetics, i.e., the competing cavity photon emission is faster than the aggregation process, or by thermodynamics, i.e., the lower polariton branch has a lower energy than the aggregated state. However, for the delayed emission case, the aggregate forms on the triplet manifold and a direct excimer to polariton transition needs to occur.

#### 3.2.1 Photochemical Reactions

A chemical reaction is defined as the breakage or formation of chemical bonds, and a photochemical reaction is defined as this happening on an electronically excited surface. The starting point of the field of polaritonic chemistry probably occurred from the study of photochemical reactions using photoswitchable molecules. Such molecules contain two chemical states, having distinct chemical and physical properties, which can switch in between each other using light stimulus.^167−169^ It was first shown that a conversion in the photoswich couple of spiropyran and merocyanine could occur when merocyanine was strongly coupled to a cavity mode.^19^ Then, the rate of the photochemical conversion and the equilibrium concentration of the photostationary state were shown to be modified.^166^ When coupling one species strongly, its excited state energy can relax to the lower polariton. The lower polariton then provides an additional relaxation route that resulted in a lower photoisomerization efficiency, phenomenology explaining the results (Figure 6c,d). It is important here to distinguish between the rate of the excited state decay and that of the photochemical transformation. An additional (nonreactive) relaxation channel gives a faster excited state relaxation, resulting in a slower macroscopically observed photochemical conversion. Later, the quantum yield of a photochemical reaction in the strongly coupled regime was examined. It was found that the photoisomerization efficiency depends on coupling and excitation conditions (Figure 6e).^117^ While the photoisomerization efficiency was about the same as in an uncoupled film when exciting the upper polariton branch and the exciton reservoir, it decreased when exciting the lower polariton branch. This dependence can be explained by the competing process between cavity photon emission from the lower polariton, which is introduced as a new decay channel, and photoisomerization itself. The decrease in the photoisomerization efficiency was more prominent for a cavity having an increased photonic contribution to the lower polariton, which supports the competition picture. In addition to the photonic/excitonic constitution of the polaritons, the energetic overlap between the polaritons and the exciton reservoir plays an important role for the relaxation dynamics. Groenhof et al. have shown that the lifetimes of the lower and upper polariton branches are limited by rapid photoemission (based on the short cavity lifetime) and the population transfer to the exciton reservoir.^118^ The population transfer strongly depends on the overlap between the polaritons and the exciton reservoir, which resembles bare molecule absorption and is quite broad in organic dyes. In order to avoid transfer from the lower polariton branch to the exciton reservoir, the energy levels need to be well separated. In addition to this, several other theoretical studies related to photochemical transformations in the strong coupling exists.^83,170−175^

#### 3.2.2 Energy Transfer

**Figure 7:**
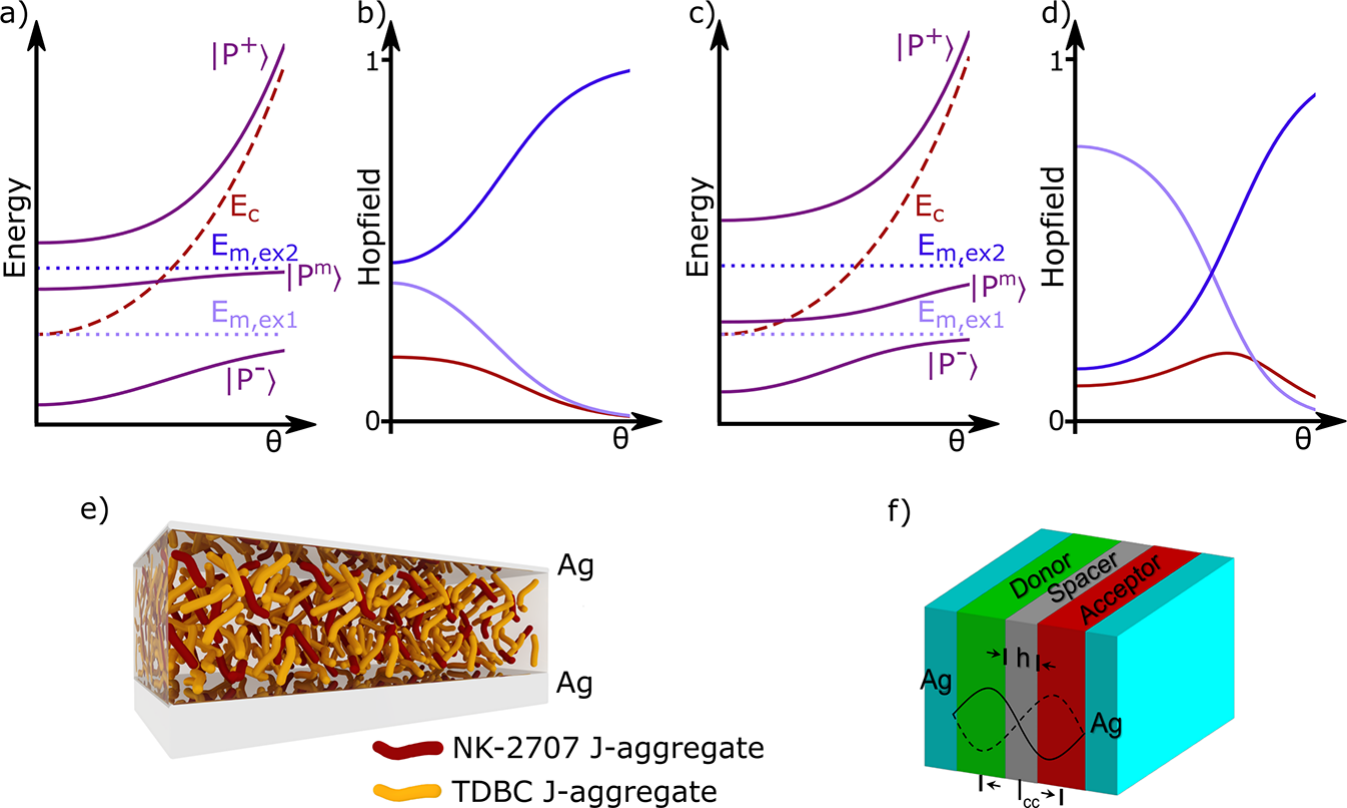
(a) Energy sketch of the dispersive behavior of the polaritons (purple) for the case that the coupling strength between the first exciton (light blue) and the cavity photon (red) is larger than the interaction of the second exciton (dark blue) with the cavity photon. (b) Contribution of the photonic (red), first excitonic (light blue), and second excitonic (dark blue) part to the middle polariton corresponding to ‘a’. (c) Energy sketch of the reverse case where the interaction between second exciton with cavity photon exceeds the interaction between the first exciton and cavity photon. (d) Contribution to the middle polariton for the case corresponding to ‘c’. The figures were obtained from a coupled harmonic oscillator model using three oscillators representing the two excitons and one photon mode. (e) Bulk donor–acceptor film inside an optical microcavity. The film is the blend of two J-aggregates TDBC (yellow) and NK-2707 (red). Here, the TDBC J-aggregate is the donor and the NK-2707 J-aggregate is the acceptor for the energy transfer reaction. Reproduced from ref (179). Copyright 2014 Springer Nature. (f) Energy transfer from the TDBC J-aggregate donor to the BRK J-aggregate acceptor, with the two dyes separated with a 75 nm spacer. Reproduced from ref (180). Copyright 2017 John Wiley and Sons.

In earlier sections, we have mainly focused on the dynamics of exciton photon coupling involving a single type of exciton species and a single cavity mode. In this section, we will focus on multiple exciton species coupled to a single photonic mode, a single exciton species coupled to multiple photonic modes, and multiple exciton species coupled to multiple photonic modes. When two exciton species with distinctly different energies strongly couple to a single photonic mode, three polaritonic states are formed (Figure 7a–d). These we denote as the upper, middle, and lower polariton.^176,177^ The upper and lower polariton mainly have an exciton contribution from the high and low energy excitons, respectively. However, the middle polariton contains contributions from both excitons. The character of the middle polariton depends upon the light–matter interaction strength of the excitons and the cavity mode. In the case of an unequal interaction strength (which is based on the oscillator strength and concentration of the molecular species), the dominant species will have the highest influence on the middle polariton energy. Furthermore, as the cavity mode is tuned, for instance by increasing *k*_∥_, the resonance conditions change and the cavity mode goes from being on resonance with the low energy exciton to being on resonance with the high energy exciton. When doing so, the fractional excitonic contributions from the molecular species to the middle polariton also change. This change means that a single cavity mode can couple to two energetically distinct excitons at different positions in the *k*_∥_ space. The excitonic contribution to the middle polariton is dominated by the molecule, which is in resonance with the cavity mode.^178^

As this middle polariton has an excitonic contribution from both excitons, it can act as a channel for nonradiative energy transfer between the two molecular species (Figure 7e).^179^ This process occurs because a simultaneous change in the contribution to the middle polariton occurs when energetically relaxing along *k*_∥_ space.At high *k*_∥_, the middle polariton mainly contains a contribution from the high energy exciton, and at low *k*_∥_ the middle polariton mainly contains a contribution from the low energy exciton. Experimentally, emission from the lower polariton (mostly containing contributions from the low energy exciton) after excitation of the upper polariton (mostly containing contributions from the high energy exciton) is used to probe the efficiency of the energy transfer event. Using this approach, the energy transfer efficiency was enhanced by a factor of 7 inside a cavity containing a bulk donor–acceptor film.^181^

Transferring the exciton energy over distances exceeding 10–100 nm is difficult. Both Förster type energy transfer and exciton diffusion are generally inefficient on these length scales. In the creation of polaritons, it is not important where in a cavity the molecules are located as long as the interaction strength between the molecules and the electric field at that position is large enough. Thus, the polaritons can be viewed as delocalized over the whole cavity. Indeed, polariton assisted energy transfer between two different molecular species, separated by a 75 nm spacer layer, was demonstrated not long after the first observed polariton assisted energy transfer event (Figure 7f).^180^ Conceptually, this work was a milestone within the field of polaritonic chemistry. This is because it demonstrated nonradiative energy transfer at distances not accessible by traditional photophysical processes. Still, as of today, energy transfer is the only example where the formation of polaritons pushes the state-of-the-art performance of photochemical and photophysical transformations.

The polaritonic energy transfer efficiency increase with red-detuning of the cavity.^182^ The length scale of polariton mediated energy transfer was later extended to the micrometer scale.^183^ At these length scales, cavity modes come close in energy in the visible part of the electromagnetic spectrum. Multiple cavity modes can therefore be coupled to a single exciton.^184−186^ Thus, this results in the formation of multiple upper and lower polariton branches, each pair corresponding to an individual cavity mode. For the case of two excitons simultaneously coupled to multiple cavity modes, the result is the formation of many middle polaritonic states, each corresponding to a cavity mode that is simultaneously coupled to both excitons.^183^ It is unclear if the increased number of middle polaritonic branches between the two excitons leads to a rise in the number of possible relaxation channels and thus enhanced efficiency of polariton assisted energy transfer. Polariton mediated energy transfer has also been extensively explored theoretically.^187−192^

Generally, the lower polariton primarily contains a contribution from the low energy exciton. However, in some circumstances (when the lower exciton has a minute transition dipole moment compared to the higher energy exciton) the lower polariton can contain a larger contribution from the high energy exciton than from the low. For such cases, the low energy exciton will be the major contributor to the middle polariton. Thus, the energetics and the direction of energy transfer are reversed.^193^ The reversal in energy transfer from the acceptor to the donor in the strong coupling regime has been explained theoretically.^187,194^

#### 3.2.3 Singlet Fission

**Figure 8:**
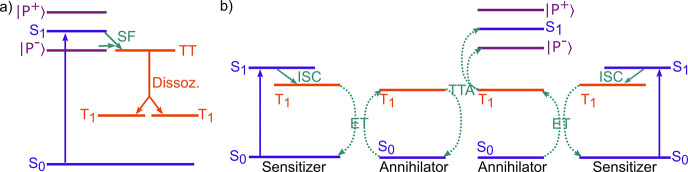
Pictorial illustration of (a) singlet fission and (b) triplet triplet annihilation. Here, TT represents the triplet pair, SF singlet fission, ET triplet energy transfer, and TTA triplet–triplet annihilation. The blue arrows represent the excitation event in a typical experiment, green arrows represent nonemissive excited state transfer events, and green dotted arrows represent intermolecular processes. Polaritonic states are shown in purple. The formation of polaritons affects the energy alignment with the triplet pair, resulting in modified kinetics of the SF and TTA processes.

The process of singlet fission converts one highly energetic singlet state to two triplet states (Figure 8a). The rate of singlet fission depends upon the energetic overlap between the singlet state and the so-called triplet pair, which can be seen as an intermediate step, as well as on molecular packing:^195−197^
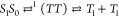
27

Theoretical studies showing an acceleration of singlet fission in the strong coupled regime exist.^198^ The rate of singlet fission can be improved in the strong coupled regime for a particular cavity energy detuning.^199^ This is because as the conversion is of an excitonic nature, the rate should inversely vary with the energy gap between the singlet state and the triplet pair (*E*_*S*1_ – *E*_*TT*_). In the strong coupled regime, the first excited singlet state hybridize to from the polaritonic states. However, triplet states remain energetically unperturbed.^200^ Two energetic possibilities of the strongly coupled system are now possible. In the case of *E*_*LP*_ > *E*_*TT*_, the rate of singlet fission increases, as the energy gap in the cavity (*E*_*LP*_ – *E*_*TT*_) decreases as compared to that of the bare film (*E*_*S*1_ – *E*_*TT*_), making the energy surfaces more isoenergetic. In the case of *E*_*LP*_ < *E*_*TT*_, the exciton reservoir remain the active state for singlet fission. The rate of singlet fission was therefore argued to remain unchanged, although it could easily be imagined that the lower polariton introduced a relaxation channel that could potentially reduce the singlet fission efficiency. Experimentally, no observations of a changed singlet fission yield in the strong coupling regime has so far been observed.^201^ Here, TIPS-pentancene was used, in which 2*E*_*T*1_ is close to *E*_*S*1_.^202^ By strongly coupling *S*_1_ to a cavity mode, the resulting energetics was *E*_*LP*_ < 2*E*_*T*1_, potentially explaining the experimental outcome.

### 3.3 Relaxation from Molecular Centered States to Polaritonic States

After discussing how delocalized polaritons when relaxing can alter the dynamics of localized events, the focus is now put on the reverse process. That is, the transfer of energy from a molecular centered state, not taking part in the light–matter hybridization event, and the lower polariton. The transition dipole moment between triplet states and the singlet ground state of organic molecules is low since it is a spin-forbidden transition. It is therefore expected to remain unperturbed inside an optical cavity. However, as the singlet state strongly couples to a photonic mode, forming polaritonic states, the energetics of the system change. The relative energy levels of the excited singlet/polaritonic and triplet states govern the rate of processes such as reverse intersystem crossing (RISC) and triplet–triplet annihilation upconversion (TTA-UC), both of which have been examined in the strong coupling regime.

#### 3.3.1 Reverse Intersystem Crossing

Reverse intersystem crossing is the process where a molecules goes from its first excited triplet state to its first excited singlet state.^203^ This process has found a large technological relevance within OLEDs, were electrical injection leads to the creation of a large amount of excited triplet states.^204−213^ Since the process of reverse intersystem crossing needs to be thermally activated, it shows a large dependency on the energy gap between the two states. The smaller the energy gap, the higher the rate. The creation of low energy polaritons therefore holds the potential to significantly enhance the rate of triplet-to-singlet-state transitions, which could be of significant technological importance. Erythrosine B was used in the first study, exploring if the RISC process can be affected by the creation of polaritonic states. This molecule exhibits both fluorescence and photosphorescence at room temperature, and it was shown that the energetics of the excited triplet state was unperturbed by strong coupling to the excited singlet state.^200^ The introduction of the polaritons led to a decreased energy gap between the triplet state and lower polariton, which was experimentally observed as a decrease in the activation barrier when an Arrhenius type of analysis. Although the analyses used when calculating the rate of RISC was quite simplistic, this first trial indicates that a direct transfer between a molecular centered and a polaritonic state is possible.

The first energy inversion of the lower polaritonic state below the first excited triplet state was reported using the molecule 3DPA3CN.^154,214^ This molecule exhibits a small singlet–triplet energy gap of Δ*E*_*TS*_ = 0.1 *eV* and shows no phosphorescence. However, no change in the rate of RISC was observed, despite the inversion of energy levels. The explanation given was based on a mismatch of the wave function overlap between the molecularly localized triplet state and the delocalized polariton (Figure 4a,b).^215^ This mismatch gives a scaling factor of the rate of relaxation from the triplet state to the lower polarity of 1/*N*. The rate of transfer from the triplet state to the exciton reservoir therefore exceeds the rate of transfer from the triplet state to the lower polariton, and no significant change in the RISC rate is observed in the cavity as compared to the bare film.

**Figure 9:**
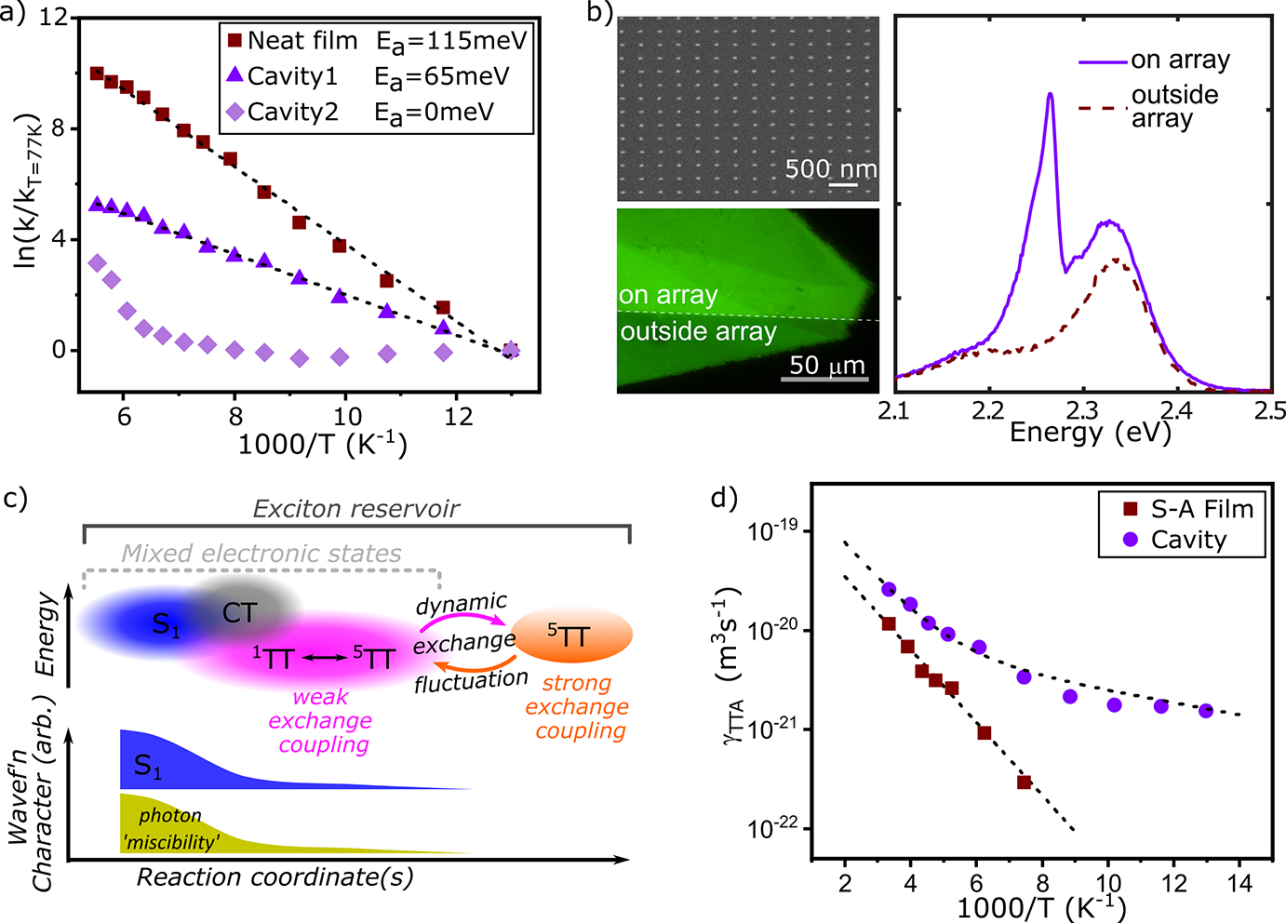
(a) Arrhenius plot with calculated activation energies. Red represent neat film, dark purple represent Cavity 1 (*E*_*T*1_ < *E*_*LP*_) and light purple represent Cavity 2 (*E*_*LP*_ < *E*_*T*1_) of DABNA-2. Reproduced from ref (217). Copyright 2021 Springer Nature. (b) Emission after triplet triplet annihilation in a tetracene crystal on top of an on resonance plasmonic array. Emission is enhanced when the system is on the array. Reproduced from ref (226). Copyright 2019 John Wiley and Sons. (c) The exciton reservoir consists of states of mostly singlet character. However, small contributions of high spin states can result in cavity-mediated triplet triplet annihilation enhancement. Reproduced from ref (227). Copyright 2020 the Royal Society of Chemistry. (d) The rate of sensitized TTA of DPP(PhCl)_2_ in the strong couple regime (purple) and in film (red). Reproduced from ref (228). Copyright 2021 American Chemical Society.

More recently, an energy inversion of the polaritonic and triplet states was achieved using the molecule DABNA-2.^216,217^ Here, the results supported a direct transition from the triplet to the polaritonic state at low temperatures. When the energy level of the lower polariton was slightly below the energy of the triplet state, the rate of transfer stays constant over a temperature range of 77–150 K (Figure 9a, Cavity 2), indicating a barrier-free RISC. Furthermore, the measured activation energy scaled with the polariton energy, giving further support for the picture of a direct transfer between states. However, in far red-detuned systems, a loss of the connection between the triplet and the polaritonic state was observed, which was explained by the authors as being due to the low excitonic character of the lower polariton.

The previously described works show contradictory behaviors. On the one side is the activation barrier for RISC reduced due to strong coupling on the one side and on the other side no change observed at all. Putting the selected molecules into focus, the difference between the works is that molecules showing an increased RISC rate exhibit phosphorescence and the molecule without a change in the rate shows no phosphorescence. The aspect of a photonically mediated transition was part of a recent theoretical work, which successfully simulated the experimental results using Erythrosine B.^218^ An additional triplet to polariton rate triggered by the coupling between the triplet and the photonic part of the lower polariton was introduced. This rate is present only for molecules with non-negligible transition dipole moments for excited triplet to ground state singlet transitions, which is the case for molecules showing phosphorescence. Or, perhaps more generally stated, when the rate of phosphorescence is on a comparable size scale to the other rates deactivating the excited state. It further depends on the energy overlap between the triplet state and the lower polariton, which in general is large due to the broad transitions of organic molecules. Although this theoretical explanation seems to be phenomenologically applicable on all three publications on the subject, more molecules need to be explored in order to conclude its validity.

#### 3.3.2 Triplet Triplet Annihilation

Triplet–triplet annihilation is the reverse reaction of singlet fission (eq 27). It involves the annihilation of two excited triplet states to generate one higher in energy excited state, which can be a singlet state (Figure 8b).^219−225^ It occurs when two molecules in their excited triplet states interact, forming what is commonly referred to as the triplet pair, which can relax to the first excited singlet state if *E*_*S*1_ < 2*E*_*T*1_. TTA was first explored in the strong coupling regime using tetracene crystals, where an enhancement of the delayed fluorescence signal was observed (Figure 9b).^226^ The efficiency of TTA is generally considered to be governed by spin-statistic. Two triplet states can in the triplet pair be combined in 9 possible ways, where 1 being of singlet, 3 of triplet, and 5 of quintet character. Here, it is only the singlet state that is of technological relevance as it can generate an emitted photon. Interestingly, it has been found that there is a connection between polaritonic and quintet states (TT^5^) in geminate TTA, using TIPS-tetracene (Figure 9c).^227^ The creation of polaritons could then be viewed as a possible means of reducing the effect of spin statistics on TTA efficiency.

If the triplet state is generated by triplet sensitization, then TTA can function as a photon upconversion mechanism. To achieve this, a sensitizer molecule must be introduced. The role of the sensitizer molecule is to absorb low energy photons, which after intersystem crossing occur at high efficiency. Triplet energy transfer to the annihilator molecule then occurs, followed by TTA and the emission of a high energy photon. A net increase in photon energy is achieved when the energy gained in the TTA step is larger than the sum of energy losses for one photon in the intersystem crossing and triplet energy transfer steps (Figure 8b). The rate of TTA photon upconversion was investigated using 3,6-bis(4-chlorobenzene)diketopyrrolopyrrole (DPP(PhCl)_2_) as an annihilator molecule and a porphyrin as the triplet sensitizer.^228^ DPP(PhCl)_2_ does not fulfill the energetic condition for TTA, but when strongly coupled to a cavity mode, the formed polaritons does so. The relative TTA photon upconversion efficiency in the system was shown to increase with the driving force for the TTA process, and the rate of TTA photon upconversion was explored as a function of temperature (Figure 9d). In the strongly coupled system, having the ability to perform exothermic TTA, did the process proceed at temperatures where no emission could be observed in the control film, in which TTA was an endothermic process. Furthermore, whereas the rate of TTA showed a considerable increase in the strong coupling regime, the natural decay rate of the triplet states was unperturbed. Thus, coupling of one molecular transition does not seem to affect the decay rate of other molecular transitions (that have very small light–matter interactions). These studies all point to the direction that a direct transition from the triplet pair to a polaritonic state is present in different molecular systems, and that it can increase the efficiency of TTA by increasing the driving force and changing the spin conservation requirement for the process.

### 3.4 Strong Coupling in Organic Electronics

The strong coupling regime can benefit organic electronics in a variety of ways. From the charge injection perspective, it has been shown that the work-function of organic materials can be altered within the strong coupling regime.^229^ This shows that the creation of polaritons could influence work-function matching and thus the need of overpotentials in devices. In organic materials, charges can be transported in the ground state (conductivity) and excited state (photoconductivity). To the surprise of many, the conductivity can be increased in a strong coupling regime. The creation of polaritonic states affects charge transfer in both the conduction band^230^ and the valence band.^231^ When light is absorbed by an organic material, the amount of free charge carriers increases, enhancing the conductivity. Further, because this effect is based on excited state processes, one would naively assume that the photoconductivity would be altered in the strong coupling regime. Indeed, changes in the conductivity has been observed for both neutral^231^ and charged (polarons)^232^ organic materials. The effect was explained phenomenologically by an altered thermalization length of the photoexcited charge carrier.

#### 3.4.1 Electrically Pumped Light Emitting Devices

Polariton research is often motivated by their interesting photonic properties, such as narrow line width emission and the possibility of thresholdless lasing. From a technological perspective, taking advantage of these features in applications requires that the excited state is reached by electron injection. The first organic light emitting diode working in the strong coupling regime was based on a TDBC emission layer.^233,234^ Although devices were less efficient in the strong than in the weak coupling regime, it marks an important step toward the utilization of organic polaritonic devices. Today, several examples of ultrastrongly coupled organic light emitting diodes exists.^159,160,235^ In the ultrastrong coupling regime, the lower polariton energy is less dependent on *k*_∥_. Thus, resulting in a more constant emission color with viewing angle, which is beneficial in real world applications. Light emitting field effect transistors also has been made to work in both the strong^236^ and ultrastrong^237^ coupling regimes. Light-emitting diodes and polariton lasers based on inorganic semiconductors are also known, but lasing is restricted to low temperatures.^238−241^

Organic materials for achieving strong exciton–photon coupling do not always produce high-performance organic electronics. To separate emission from a strongly coupled organic dye from the charge recombination process, hybrid organic–inorganic light emitting devices can be made. In these devices, an inorganic material functions as the electron–hole recombination site, and the energy is then transferred to an organic dye in the strong coupling regime. The two materials can either be located in the same cavity,^140,242^ or in two distinct but coupled cavities.^243^

#### 3.4.2 Organic Photovoltaics

In organic photovoltaics (OPV), excitons are formed upon illumination of light. To form a current, the exciton must first diffuse to charge transfer states located at the interface between electron-donating and an accepting materials. If the binding energy is not too high, electrons and holes can then separate to form free charge carriers, which can diffuse to the electrodes and be recorded as a current. Limitations in OPV technology include voltage losses due to the relatively large reorganization energy in organic materials and the short exciton diffusion length of organic materials. The latter is often mitigated by the blending-phase separation process performed in the so-called bulk heterojunctions. The organic materials used in OPV’s are efficient light absorbers, and the size scale of devices is compatible with forming Fabry–Perot cavity modes in the visible regime of the electromagnetic spectrum. It is therefore possible to form strongly coupled systems using functional OPV devices. As a side effect, there is a high likelihood of serendipitous strong coupling in the OPV literature. This because the refractive index mismatch of structures of appropriate size is enough to reach the strong coupling regime.^59−62^ Polaritons can bring two benefits to the field of OPVs. They can reduce the reorganization energy, which can be experimentally viewed as a red-shifted absorption edge.^244^ Their delocalized nature can also be used as a means to transport excited state energy over large distances, thus increasing the apparent rate of exciton diffusion and reducing the need to construct bulk heterojunctions.

In the first deliberately made OPV in the strong coupling regime, a reduction of the reorganization energy was targeted.^245^ In an organic photodiode, based on a phthalocyanine (SnPc):C_60_ bulk heterojunction, the SnPc ultrastrongly coupled to a cavity mode. The formed lower polariton then provided a red-shifted response, which can be viewed as a reduction of the reorganization energy of the SnPc. Photodiodes working the near-infrared regime of the electromagnetic spectrum has also been realized by coupling fluorescent carbon nanotubes.^246^ The concept was expanded to organic solar cells. A reduction in the energy loss during photon conversion (*E*_*Opt*_ – *qV*_*OC*_) and steepening of the absorption edge in the strong coupling regime were observed.^247^ This using SubNc/Cl_6_PhOSuPc and SubNc/C_60_ layered heterojunctions, being thin enough for an efficient diffusion of excitons to the interfacial charge transfer states but still thick enough to drive the system into the strong coupling regime. Here, *E*_*Opt*_ is the optical band gap, *V*_*OC*_ is the open circuit voltage of the device under 1 sun illumination, and *q* is the elementary charge. The optical band gap decreased, while the open circuit voltage of the device remained unaffected in the strong coupling regime. This was because the charge transfer state was energetically unaffected due to its small transition dipole moment. The voltage loss decreased up to 60 meV in the strong coupling regime. The common element of these two studies is that energy from the energetically relaxed lower polariton can relax to the interfacial charge transfer states. Thus, enabling an effective red-shift of the optical response is enabled due to the low reorganization energy of polaritons.

Recently, it was proposed that the delocalized feature of the lower polariton also can assist in the transfer of energy to localized charge transfer states.^248^ Thus, opening up the possibility for using polaritons, formed by wave function mixing of excitons to the vacuum field, instead of physically blending materials to form bulk heterojunctions. A layered heterojunction that is inefficient at transporting exciton energy to the interfacial charge transfer states was examined. It was found that when the system entered the strong coupling regime, the excited state lifetime was reduced by about 1 order of magnitude, while the external and internal quantum efficiencies increased by about 1 order of magnitude. Thus, suggesting an energy funneling effect when delocalized polaritons relax to localized charge transfer states. Indeed, the transfer from the lower polariton to interfacial charge transfer states has now been observed also by transient absorption.^249^ The enhancement of charge transfer in organic photovoltaic under strong coupling has been well studied theoretically,^189,250^ and so has exciton funneling from the delocalized polariton toward reactive sites.^251^ Exciton transport behavior of organic polaritons has furthermore been studied with microscopy techniques.^252−254^

## 4 Plasmonic and Open Cavities

### 4.1 Plasmonic Nanoantennas

Plasmonics paves the way toward the strong coupling regime by confining light at the subwavelength scale using metallic nanoresonators.^255,256^ The main advantage of plasmonic cavities over Fabry–Perot ones is that they possess small subwavelength mode volumes, which allows the strong coupling regime to be achieved even on a single-molecule level and at room temperature. However, the involvement of free carriers in the metallic components inevitably entails Ohmic losses and a strong reduction of the cavity quality factor. Recent works have shown the possibility of achieving strong coupling with organic molecules using dielectric nanoresonators, where Ohmic losses are absent.^257−263^ This Review further focuses on plasmonics resonators.

In the early works preceding the observation of Rabi splitting in plasmonic systems, the coupling of molecular or excitonic resonances to plasmonic nanostructures was usually considered as a “coherent” coupling effect.^264−269^ Such an effect in the weak interaction regime is characterized by a Fano-like profile of the scattering spectrum (an asymmetric line-shape caused by two resonances of different line width).^270,271^ However, due to the presence of two peaks in the scattering, this regime can potentially be confused with the true Rabi splitting, which can be reliably recognized only when the splitting substantially exceeds the full-width at half maxima of polaritonic bands or by signs of polariton splitting in absorption or photoluminescence spectra.^74,272−274^ In the coherent coupling regime, the scattering dip is a result of destructive interference between the plasmon and excitonic resonance in the far-field.^275^ Unlike in the strong coupling regime, when the two peaks corresponding to the polariton modes split at a value exceeding the polariton line width γ_m_ + γ_pl_, in the coherent coupling regime this splitting can still be much smaller than the plasmon line width 2γ_pl_. However, only strongly coupled systems support an ultrafast energy exchange between two polariton states (Rabi oscillations), which, for example, was observed in real-time for a plasmon-exciton (plexcitonic) system on a ∼10 fs time scale.^127^

To the best of our knowledge, the first demonstration of plexcitonic strong coupling was performed at room temperature for an ensemble of organic molecules (J-aggregate) covering a silver hole array.^276^ After that, a large number of both theoretical and experimental works reported similar values of Rabi splitting in different systems consisting of metallic and molecular components.^277−283^ From the biochemistry perspective, it is quite interesting that plexcitonic strong coupling in light-harvesting complexes (namely in purple bacteria) is sensitive to the specific molecular organization and the protein coverage of the pigment molecules.^284^ Among numerous manifestations of plexcitonic strong coupling, here, we mention several especially striking examples: ultrafast manipulation of strong coupling in molecular aggregate hybrid nanostructures,^285,286^ ultrafast energy transfer between molecular assemblies and surface plasmons,^287^ and coherent emission from a disordered molecular system.^288^ It is important to note that the plexcitonic strong coupling has been obtained in a great variety of systems with different geometries and materials of both excitonic and plasmonic components, including metallic nanoshells and core–shells,^289−291^ sharp tips and platelets,^292,293^ nanostars,^294,295^ and nanostructures of higher complexity.^296^ Some works confirmed the strong coupling regime by observing signs of polariton formation not only in the scattering spectra but also in the photoluminescence,^291,297^ which as discussed above provides additional evidence.

For any geometry, Ohmic losses reduce the cavity quality factor, however, arranging plasmonic nanoparticles into periodic arrays offers a way to increase the quality factor through the Fano interference of the localized plasmons with the diffractive modes.^298−301^ Owing to surface lattice resonances plasmonic arrays combined with excitonic layers enable the boost of the excitonic emission to the level of lasing, which has been demonstrated by several groups in the near-IR and visible ranges using a plasmonic array together with organic dyes^18,302−308^ (more works can be found in ref (309)). Rabi splitting in plasmonic diffractive arrays coupled to various excitonic layers (organic dyes or quantum dots) has been observed in several configurations.^310−314^ An attractive advantage of this type of plasmonic nanoantennas is the ability to change the effective mass and the composition of the polariton state by tuning the lattice geometric parameters.^311,312^ In contrast to individual nanoantennas, exciton-polaritons in plasmonic arrays can demonstrate a delocalized spatial behavior, which can be used for coherent energy transfer on the scale of the lattice.^315^ Owing to the strong coupling to surface lattice resonances, a high degree of spatial coherence can be obtained even for very exciton-like (80%) polaritons.^316^ Such a sharing of the photonic coherence by the excitons is a key step to switch from ordinary lasing to Bose–Einstein condensation of exciton-polaritons^18,317^ (see details in Section 5). More details about plexitonic strong coupling in the diffractive arrays can be found in references (75, 300, and 318).

#### 4.1.1 Single Nanoantennas

The observations of the plexcitonic strong coupling discussed above were realized in an ensemble of plasmonic nanoparticles. However, such ensembles are not very well suited to studying single molecules or quantum dots. In order to better control the coupling with single emitters and to reduce the contribution of various ensemble mechanisms to the plasmonic resonance broadening, researchers turned their interest toward the coupling of excitonic materials to *single* nanoantennas. The pioneering observations of plexcitonic strong coupling in single plasmonic nanoantennas interacting with J-aggregates were reported by Schlather et al.^319^ using plasmonic dimers and Zengin et al.^320^ using individual silver nanorods. Later, Zengin et al.^321^ reached the strong coupling regime using an individual silver nanoprism coupled to J-aggregates and estimated from the experimental data that the number of excitons involved in such a plexcitonic coupling is no more than approximately 80. Furthermore, the nanoprism used in this work compresses the mode volume enough to achieve comparable to photonic crystals or microring resonator cavities strong coupling figure-of-merit . In later works by Wersäll et al.,^322,323^ the plexcitonic strong coupling in a single plasmonic nanoprism embedded in J-aggregates was demonstrated not only by splitting in the dark-field scattering spectra but also by temperature-dependent photoluminescence spectroscopy.

Nanoparticles of several tens of nanometers in size, on the one hand, create a large electric field enhancement near the surface; but on the other hand, as the light penetration depth in the metal is comparable to the nanoparticle size, most of the mode energy is concentrated inside the particle.^91,321^ That is why plasmonic dimers have become so popular–they are able not only to enhance the electric field in the gap region but also to reduce the mode volume by localizing the mode within the gap region both inside and outside of the metal.^274,319,324^ The bowtie-shaped dimers are among the most effective and convenient gap nanoantennas from an experimental point of view. In the gap region, they are essentially metallic nanomirrors with a width of about the size of the gap and are arranged perpendicular to the substrate. Such a configuration provides exceptionally high electric field enhancement in the gap while normal incident light is used for excitation. Moreover, this configuration is highly selective to light polarization, with the light polarized along the bowtie axis providing the highest enhancement. Plasmonic nanocavities supporting gap plasmon modes can also be realized by using the nanoparticle-on-mirror strategy. These can be implemented using various types of metallic particles, including nanocubes, nanorods, nanoprisms, nanospheres, etc., and will be discussed in detail below.

#### 4.1.2 The Single-Emitter Limit

Different realizations of plexcitonic strong coupling with single plasmonic nanoantennas paved the way to approaching the single-emitter limit. Its achievement opens up new opportunities in the creation and control of quantum states at the single-photon level, which has great prospects for the creation of single-photon sources,^325^ quantum optical circuits,^326^ and ultralow-power lasers.^327,328^ In contrast to the molecular aggregates with a large number of emitters, single molecules have transition dipole moments of a few Debyes, which therefore requires even stronger compression of the field in a nanocavity to reach the strong coupling regime. Quantum dots, usually having an order of magnitude larger transition dipole moments,^318^ in this sense are more promising and convenient as a platform for the realization of strong coupling with single emitters.

The main experimental challenges in reaching the single-molecule strong coupling limit are 2-fold: first, on the nanocavity side, one needs to ensure strong enough confinement of the electromagnetic field without substantially increasing the losses; second, on the molecule side, one needs a molecule with a strong enough transition dipole moment, which could physically fit inside the nanocavity and ideally be oriented along the direction of the maximal field. Moreover, the plasmonic and molecular resonances must be spectrally matched. Such a scenario is rather hard to fulfill but, on a general note, requires a combination of some plasmonic nanogap cavities (such as plasmonic dimers or particle-on-mirror) with strongly resonant organic chromophores. Remarkably, the conditions for reaching the single-molecule strong coupling regime are similar to those for the optimal configuration of surface-enhanced (resonant) Raman scattering (SERS). Therefore, it is probably not very surprising that single-molecule strong coupling and single-molecule SERS were realized in similar setups, and in general, these disciplines are closely related and develop in parallel nowadays, although the latter is considerably more mature.^329^

**Figure 10:**
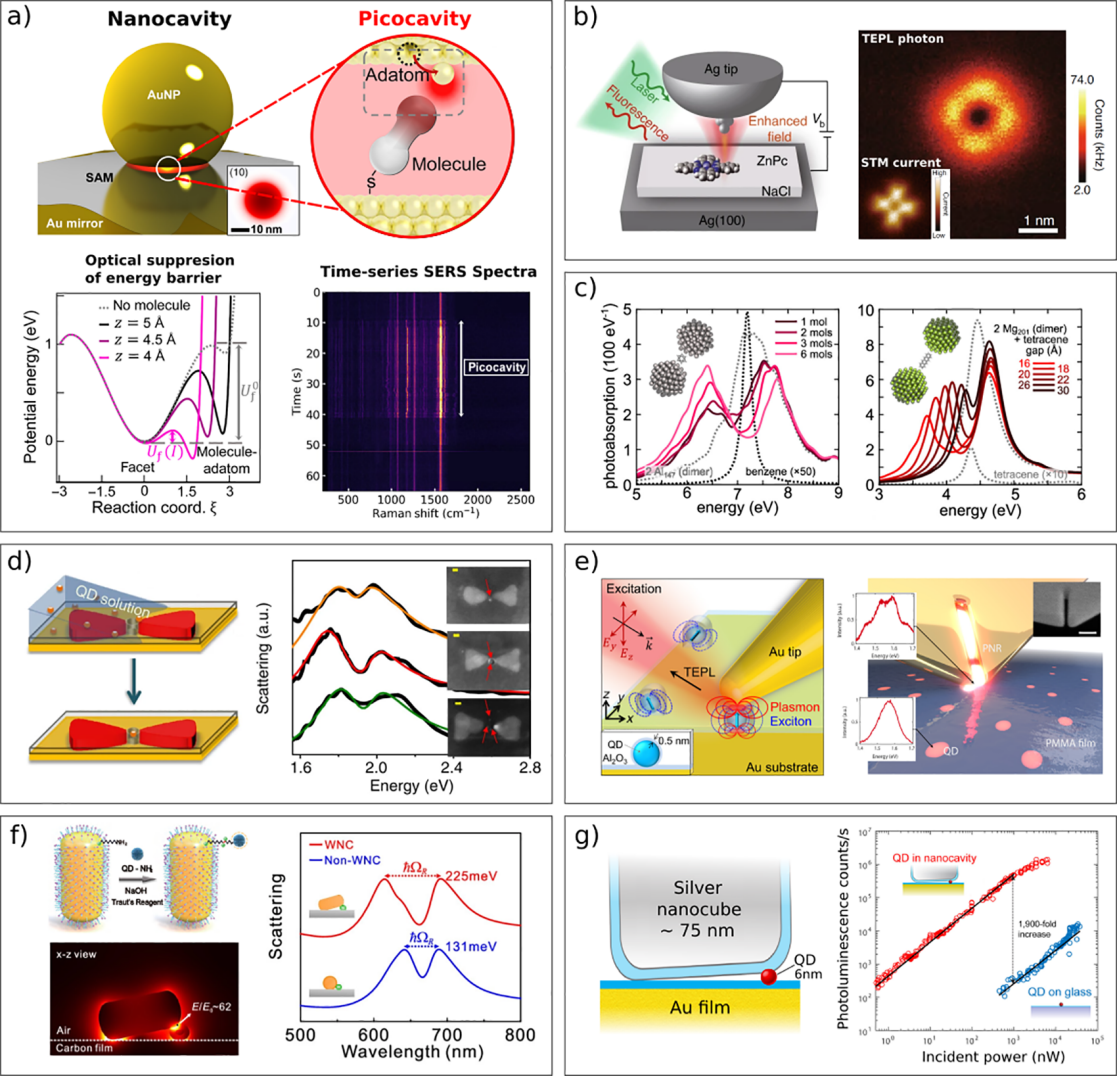
Single-emitter Rabi splitting at room temperature. (a) Schematic of a nanometer-thick self-assembled monolayer (SAM) of molecules in the gap of a plasmonic nanocavity with nanoparticle-on-mirror geometry (top left). Scheme of a picocavity with the optical field attracting an adatom to the molecule tip (top right). Simulated energy of picocavities versus reaction coordinate of adatom when molecule tip-adatom separation *z* decreases by light (solid) and when there is no molecule (dashed), showing a reduced energy barrier for adatom extraction (bottom left). Time-series of surface-enhanced Raman scattering spectra showing the lifetime of a picocavity by new intense vibrational modes appearing (bottom right). Reproduced from ref (333). Copyright 2022 AAAS. (b) Schematic of scanning tunneling microscopy setup with the atomistic protrusion at the silver tip, giving the enhancement of photoluminescence from a molecule under the tip (left). Simultaneously recorded tip-enhanced photoluminescence photon image and scanning tunneling microscope photon image (inset) of a single zinc phthalocyanine molecule on three-monolayer-thick NaCl/Ag(100). Reproduced from ref (334). Copyright 2020 Springer Nature. (c) *Ab initio* calculations of photoabsorption spectra of Al_147_ dimers with a 10 Å gap coupled to a various number of benzene molecules showing the ultrastrong coupling even for the single molecule (left). The same for Mg_201_ dimers with a single benzene molecule for various dimer gaps (right). Reproduced from ref (337). Copyright 2022 American Chemical Society. (d) Schematic of the interfacial capillary force-assisted method for positioning quantum dots in the gap of bowtie structures (left). Scattering spectra of silver bowtie antennas with one, two, and three CdSe/ZnS quantum dots in the gap shows record values of Rabi splitting (right). Reproduced from ref (339). Copyright 2016 Springer Nature. (e) Schematics of a scanning probe microscopy setup to study quantum dots in the strong coupling regime (left) and slit-like scanning probe combining the near-field spectroscopy technique with the ideas of gap nanoantennas (right). Reproduced from refs (344) and (345). Copyright 2018 AAAS and 2019 AAAS. (f) Schematic (top left) of the self-positioning of quantum dots at the sharper corners near the ends of gold nanorods, where the electric field (bottom left) and Rabi splitting (right) are maximized. Reproduced from ref (346). Copyright 2022 American Chemical Society. (g) Schematic of a single quantum dot in the gap between a silver nanocube and a gold film (right). The location of the quantum dot near the corner of the nanocube, where the field enhancements are the largest, gives a 1900-fold increase of the room-temperature emission (right). Reproduced from ref (348). Copyright 2016 American Chemical Society.

Following this line of arguments, probably the first work noticing the relevance of SERS to single-molecule plexcitonic strong coupling was the report by Itoh et al. in 2014,^330^ where Rhodamine-6G molecules in the nanogap of a silver nanoparticle dimer were studied by means of Raman and dark-field scattering. Later, record-high numbers in terms of the plasmonic gap miniaturization were set in a series of works using nanoparticle-on-mirror geometry. First, for a gold nanosphere on a gold mirror forming a 0.9 nm gap with ultraconfined mode volume (approximately 40 nm^3^), Chikkaraddy et al. demonstrated the plexcitonic strong coupling down to a single molecule of methylene blue placed in the gap and oriented normal to the metal surface using a barrel-shaped nonresonant host molecule.^331^ Notably, the dark-field scattering spectra clearly revealed the effect of the transition dipole moment orientation, as a pronounced Rabi splitting was observed only when molecules were oriented perpendicular to the metal surface. Based on a similar nanoparticle-on-mirror geometry (Figure 10a), the formation and disassembling of atomic defects were demonstrated in the gold surfaces forming the 0.9 nm gap by sufficiently strong laser irradiation.^332^ Such defects were stable at cryogenic temperatures and could localize light to volumes below 1 cubic nanometer and therefore provide an extreme field enhancement (∼10^6^). These “picocavities” on the gold surface were formed due to the movement of the so-called adatoms. The strong optical field gradient in picocavities is able to modify the Raman selection rules and therefore can drastically affect the spectra of molecules residing in the gap. Thus, the emergence and disappearance of picocavities can be observed by monitoring the time-series of SERS spectra. Importantly, the extraction of an adatom forming the picocavity by illumination occurs only in the presence of molecules in the gap. Interactions between a polarizable atom in a molecule and a metallic atom create strong optical forces that are able to rearrange the material interface. The external illumination gives rise to the local polarization of the molecule and electrons in the metal, which reduces the energy barrier for adatom extraction and thus binds the molecule to the metal (Figure 10a). This picture rules out the photothermal mechanism of adatom formation.^333^ The bottom right panel of Figure 10a shows new intense vibrational modes of a molecule caused by picocavity formation. The intensities and frequencies of the vibrations change due to fluctuations in the single adatom–molecule bond. In general, strong field gradients provided by picocavities may pave the way toward resolving the dynamics of individual bonds within molecules. Plasmonic nanocavities with atomic defects or protrusions can also be useful for single-molecule photoluminescence subnanometre imaging. The tip-enhanced photoluminescence (TEPL) technique can provide a similar resolution (∼8 Å) as a scanning tunneling microscope can have (Figure 10b).^334^

As described above, single-molecule strong coupling is challenging to realize experimentally. Thus, a considerable effort to better understand single-molecule plexcitonic systems is attempted theoretically through numerical simulations.^89,335,336^ In particular, although single-molecule SERS and single-molecule strong coupling have been experimentally realized, reaching the *ultrastrong* coupling regime with a single molecule remains challenging. To fill this gap, it has been shown recently by time-dependent density functional theory calculations that a single organic molecule can reach ultrastrong coupling with a plasmonic dimer consisting of a few hundred atoms^337^ (Figure 10c). Experimental realization of such a strategy remains to be demonstrated and possibly could involve electron energy loss spectroscopy, as suggested previously.^338^

In parallel with single-molecule strong coupling, great progress toward single-emitter plexcitonic strong coupling was achieved using individual colloidal quantum dots. As mentioned above, quantum dots possess substantially higher transition dipole moments in comparison with molecular emitters. Therefore, single-emitter strong coupling, in this case, does not necessarily require enormous compression of optical modes and alignment of the transition dipole moment with the electromagnetic field. Pioneering works reporting such a strategy include Santhosh et al.^339^ and Hartsfield et al.^340^ Hartsfield et al. used a gold nanosphere on a glass substrate coupled to a single CdSe/ZnS quantum dot but did not obtain a pronounced Rabi splitting. Santhosh et al. managed to place a single CdSe/ZnS quantum dot in the 20 nm gap of a bowtie silver nanoantenna, using a polymeric mask defined by electron beam lithography and an interfacial capillary force, and achieved a Rabi splitting of up to 240 meV (Figure 10d). To our knowledge, this result is a record one for single-emitter plexcitonic strong coupling to date. The quantum nature of the emission from this system was confirmed recently by second-order photon correlation measurements.^341^ Also, the contribution of dark plasmon modes in the plexcitonic strong coupling using this system was revealed recently by means of electron energy loss spectroscopy.^342^

After these initial works, strong coupling between a single quantum dot and a gap plasmon was realized in different configurations of nanogaps. Leng et al. observed weak coupling (the Purcell effect), intermediate coupling (Fano interference), and strong coupling (Rabi splitting) at room temperature for a single colloidal quantum dot in the gap between a gold nanoparticle and a silver film.^343^ Groß et al. and Park et al. combined the scanning probe microscopy technique with the nanogap formation between a scanning tip and a gold mirror,^344^ or at the apex of slit-like scanning probe,^345^ which enabled better control and imaging of the strong coupling between the gap plasmons and single quantum dots (Figure 10e). Li et al. used the surface functionalization of gold nanorods with cetyltrimethylammonium bromide (CTAB) molecules to provide better control of the position, spacing, and quantity of the quantum dots involved in the coupling.^346^ The advantage of this approach is in the self-positioning of quantum dots at the sharper corners near the ends of the nanorods, where the electric field and coupling strength are maximized. It is based on the fact that the fewest CTAB molecules attach at the sharper corners near the ends of the nanorods, resulting in higher possibilities for binding the surface-functionalized quantum dots on these sharper corners (Figure 10f). Similar nanogap structures were also used to enhance the brightness of single-photon emission from nitrogen-vacancy centers in a diamond^347^ or to increase room-temperature single-photon emission from quantum dots^348^ (Figure 10g). More details on plexcitonic strong coupling applications and the most complete list of different plexcitonic systems can be found in recent reviews that can be found in references (349−353).

### 4.2 Plexcitonic Photophysics and Photochemistry

As we discussed in Section 3.2.1, strong coupling of molecules with light in a cavity can modify photochemical reactions. Pioneering experiments^19,166^ in this field used metallic cavities since they are easier to fabricate, and strong coupling with molecules at room temperature does not require a high-Q cavity mode. Later on, to reach the single-molecule limit, or at least to work with a much smaller number of molecules, single plasmonic nanoantennas were used,^354^ allowing, on the one hand, to achieve a smaller mode volume required for the strong coupling with a small number of molecules, and on the other hand, providing greater access to monitor and control the system. Some plasmonic nanoantennas provide an additional advantage over microcavities or plasmonic nanoparticle arrays: they can be chemically synthesized in large numbers and exist as colloidal suspensions in a solution.^355,356^ It should be noted that contemporary experiments in this field are aimed at proving the principle of polariton photochemistry or photophysics and not necessarily at obtaining the best characteristics of the corresponding phenomena. In this regard, the use of plasmonic nanoparticles may influence various photochemical effects, even in the weak coupling regime, through a range of mechanisms. For instance, if one needs to speed up a reaction, plasmonic nanoparticles can be used as sources of hot electrons (see the review found in ref (357)). The way chemical reactions can be slowed is highly dependent on their mechanisms. For example, the suppression of photobleaching of organic chromophores observed in ref (354). in the strong coupling regime can be alternatively achieved by several other methods, ranging from a straightforward use of oxygen scavenger reagents,^358^ to quenching of long-lived triplet states,^359−361^ and surface-enhanced spontaneous emission.^362−364^ For the sake of clarity, we stress that in this section, we will focus specifically on the role of plexcitonic strong coupling in photophysics and photochemistry.

**Figure 11:**
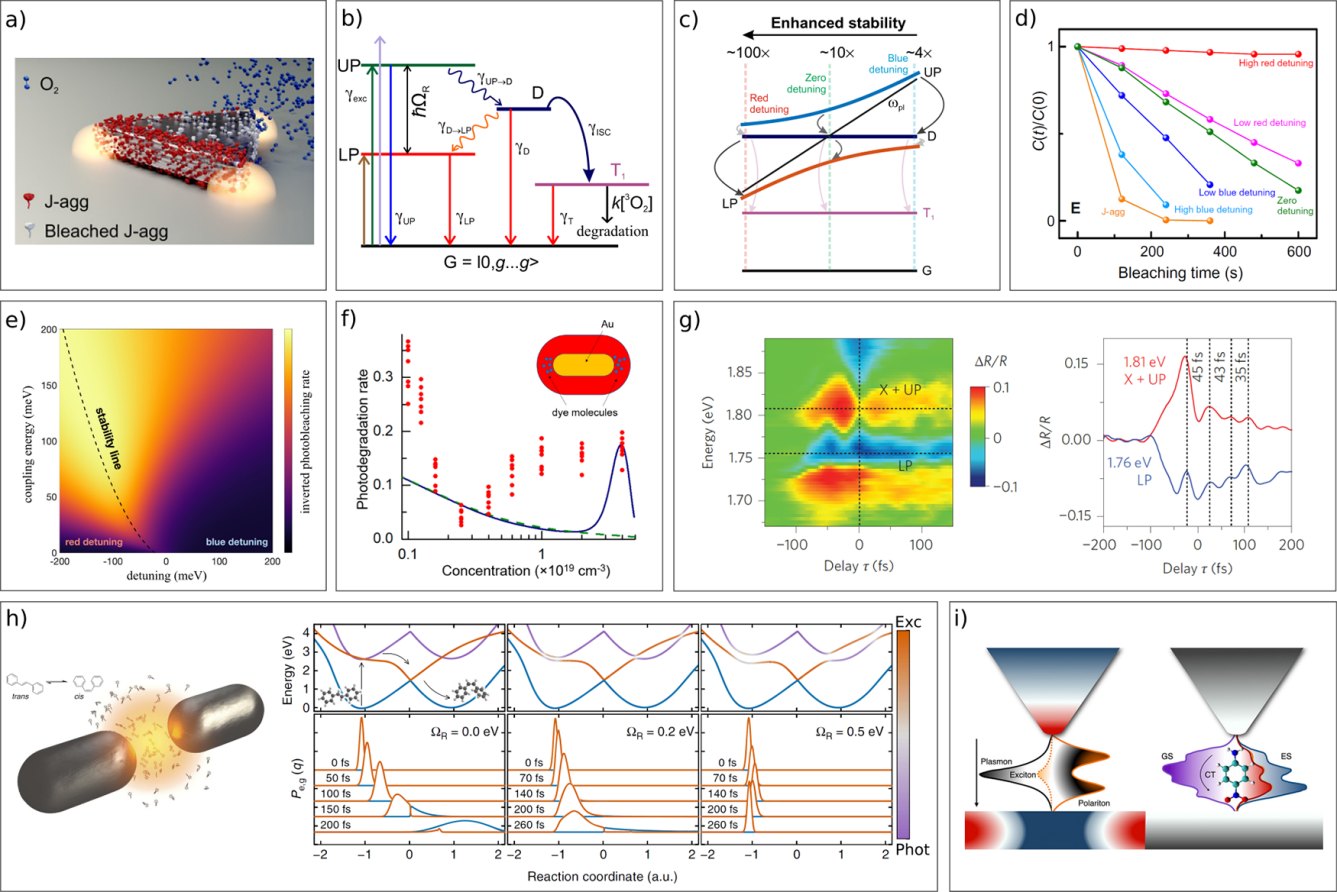
Plexcitonic photochemistry and photophysics. (a) Schematic of the photobleaching reaction of J-aggregates strongly coupled to a silver nanoprism. (b) Photobleaching mechanism of a strongly coupled system and transitions between various states: polaritons provide a fast relaxation from the excited to the ground state, bypassing the long-lived triplet. (c) Schematic of the photobleaching process as a function of plasmon-exciton detuning. The transparency of arrows shows high/low probability of the corresponding transition, demonstrating that red-detuned particles are more stable than blue-detuned ones. (d) The relative change in the concentration of active organic molecules for various plasmon-exciton detuning, compared to the uncoupled bare J-aggregates. (a)-(d) are reproduced from ref (354). Copyright 2018 AAAS. (e) Inverted population of the triplet state showing the optimal balance between the detuning and coupling energy for which the photobleaching suppression is maximized (the stability line). Reproduced from ref (366). Copyright 2020 American Chemical Society. (f) The photodegradation rate of dye molecules vs molecular concentration. The red dots are experimental data obtained from the different samples. The blue and green are full and simplified theoretical curves, respectively. Reproduced from ref (368). Copyright 2020 the American Chemical Society. (g) Coherent ultrafast manipulation of the coupling energy in strongly coupled J-aggregate/metal hybrid nanostructures by controlling the exciton density on a 10 fs time scale. Reproduced from ref (127). Copyright 2013 Springer Nature. (h) The left panel shows a schematic of molecules coupled to a plasmonic nanocavity and sketch of photoisomerization reaction between a trans- and cis-isomer. The right block of the six panels shows a calculation of the photoisomerization suppression under strong coupling for single molecules. The top row in it shows how the formation of polaritons provides new minima in the potential energy surfaces and even a barrier at larger coupling strength. The bottom row shows the corresponding trapping of the nuclear wavepacket, which means that the photochemical reaction is slowed down. Reproduced from ref (170). Copyright 2016 Springer Nature. (i) Coupled-cluster calculation of plexcitonic strong coupling reveals the modification of the ground-state (GS) electronic density caused by the molecule-plasmon interaction. The direction of charge transfer (CT) is along the arrow in the right panel. Reproduced from ref (388). Copyright 2021 the American Chemical Society.

One of the early experiments in the field of plexciton-polariton photochemistry was carried out by Munkhbat et al.^354^ The experiment demonstrated a beneficial impact of plexcitonic strong coupling on one of the most important classes of photochemical processes–the photo-oxidation (photobleaching) reaction (Figure 11a). Its mechanism involves photodynamic interactions between the excited triplet state of dye molecules and atmospheric triplet oxygen (^3^O_2_). Due to the long lifetime of the triplet state, the excited molecules have a higher probability of interacting with environmental oxygen and thus undergo photobleaching. The interaction with a cavity mode can significantly change the relaxation pathways and accordingly photobleaching in both weak coupling (Purcell regime) and strong coupling (Rabi splitting). However, owing to the delocalized and coherent nature of polaritons, only in the strong coupling regime does the effect become collective, and the relaxation pathways can be modified to a very large extent. The formation of polaritons opens up a new relaxation pathway that avoids falling from the excited singlet state to the long-lived triplet state. Due to the partial plasmonic nature of polariton states, their lifetimes are much shorter (∼10 fs) than the typical singlet–triplet transition rate (γ_*ISC*_ from ∼10 ns to ms), which provides a fast relaxation from the excited to the ground state, bypassing the triplet (Figure 11b). To make this mechanism most effective, one needs, on the one hand, to maximize the relaxation rates from the upper polariton to the ground state (γ_*UP*_) and from the incoherent state (exciton reservoir) to the lower polariton (γ_*D*→*LP*_); on the other hand, one needs to minimize the relaxation from the upper polariton to the exciton reservoir (γ_*UP*→*D*_) and the resonant excitation of the upper polariton itself (γ_*exc*_). Based on this, one can expect that red-detuned particles, owing to the reduced plasmonic component of the upper polariton, give lower γ_*exc*_ and higher γ_*D*→*LP*_; while blue-detuned particles, vice versa, provide higher γ_*exc*_ and lower γ_*D*→*LP*_ (Figure 11c). Note that this effect does not require minimization of the plasmonic losses. On the contrary, due to them, the lifetime of the excited state is significantly reduced, which makes it possible to avoid falling into the triplet state and the corresponding degradation of the molecules. However, too high plasmon losses are also undesirable, since instead of the strong coupling regime they will quench the photoluminescence of dyes into the metal.

The described mechanism of dye stabilization was demonstrated by using a set of separate plasmonic silver nanoprisms providing different initial values of strong coupling to excitons in a J-aggregate (Figure 11a). A 100-fold stabilization of organic dyes was shown for the red-detuned nanoparticles (Figure 11d). Based on the observation that strongly coupled molecules are significantly more stable than uncoupled ones, the authors concluded that the role of the hot-electron effects in this case is negligible. Indeed, hot electrons should additionally destabilize the molecules, and for the relatively large size of nanoparticles used in this experiment (∼100 nm) hot electrons are more likely to thermalize inside the metal particle before they reach the surface.

A theoretical description of the observed effect on the basis of pioneering works by Agranovich et al.^105,365^ and Litinskaya et al.^106^ was also presented. In a later theoretical study,^366^ the optimal balance between the values of red detuning and Rabi frequency for which the photobleaching suppression is most pronounced was found (see the stability line in Figure 11e). Subsequently, similar effects were demonstrated experimentally with plasmonic nanoparticles and Fabry–Perot cavities strongly coupled to various excitonic systems. A reduction of photodegradation of an organic semiconductor polymer sandwiched between silver mirrors, providing a giant Rabi splitting of ∼1.0 eV, was observed^367^ for a very important material for organic photovoltaics −2,5-poly(3-hexylthiophene) (P3HT). Normalizing the reduction in the oxygen flow caused by the top silver mirror, the authors obtained a 3-fold reduction of the photodegradation rate.^367^ Furthermore, enhanced delayed fluorescence was observed in tetracene single crystal strongly coupled to plasmonic nanoparticles arrays.^226^ The resonance behavior (Figure 11f) with the dye molecule concentration was observed for the photobleaching rate of the dye molecules strongly coupled to gold nanorods.^368^

From the photophysics side, Vasa et al.^127^ observed for the first time real-time ultrafast Rabi oscillations in a plexcitonic strong-coupling system and demonstrated coherent manipulation of the coupling energy by controlling the exciton density on a 10 fs time scale (Figure 11g). Ultrafast pump–probe studies reported a femtosecond energy transfer between excitons and plasmons,^355^ as well as the relaxation pathways in plexcitons.^136,369,370^ Particularly, Finkelstein-Shapiro et al.^136^ showed that the plexcitonic dynamics beyond a few femtoseconds must be considered in terms of hot electron distributions instead of lower and upper polariton branches. Using femtosecond-transient absorption spectroscopy, Park et al.^370^ demonstrated that due to the additional energy transfer channels both the upper and lower polaritons have shorter lifetimes than the bare excitons in a plexcitonic system consisting of a perovskite and a plasmonic nanoparticle lattice. A similar result was observed recently for the molecular photoswitches strongly coupled to anisotropic plasmonic nanoantennas.^371^

Strong coupling can also provide protection of emitters from quenching even in close proximity to a metal nanoparticle,^372^ giving rise to the “quenching of quenching”. Unlike isolated nanoparticles, which usually quench emitters by their decay at distances less than 10 nm, plasmonic nanocavities, owing to the mode hybridization, suppress the emitter’s decay into nonradiative channels and promote the re-emission of its energy.

Regarding the interpretation of the photoluminescence spectra of the polariton system, there were several suggestions about the nature of the high-energy emission peak. In some works it was concluded that it corresponds to the upper polariton emission,^373^ while others argued that it arises from the uncoupled molecules^105,106^ or dark polaritons.^374^ For the case of plexcitons, the works by Wersäll et al.^322,323^ contribute to this discussion by performing correlative dark-field and photoluminescence spectroscopy measurements on the same individual plasmon-molecule hybrid nanostructure. The unusual temperature dependence of polaritonic spectra helped the authors to attribute the lower energy photoluminescence peak to the lower polariton, whereas the higher energy peak was assigned to uncoupled molecules and incoherent states.^323^ This is in agreement with observations made in organic microcavity polaritons.^112^

We now turn our attention to the theoretical progress in organic polaritons, specifically plexcitons. First theoretical studies of exciton-polaritons did not address specifically the regime of plexcitonic strong coupling but rather focused on more general models, mostly Jaynes-Cummings or Tavis-Cummings, considering single-mode lossless cavities and treating organic molecules as two-level systems.^13^ Such an approach suffices to estimate the Rabi splitting but does not allow calculation of photochemical processes that require taking into account the internal molecular degrees of freedom. The first attempts to account for the rovibrational molecular states were based on the usage of effective decay and dephasing rates.^375,376^ Subsequently, a number of works^38,82,83,170,173,174,377−388^ based on microscopic theories of organic molecules and their strong coupling to cavity modes that contain almost full descriptions of nuclear, electronic, and photonic degrees of freedom came out. Some of these works predict not only a modification of molecular structure in the strong-coupling regime^82,377,378^ but also became the first works explaining the influence of strong-coupling on photochemical reactions.^83,170,379^

In particular, Feist and collaborators predicted the suppression of photoisomerization reactions for molecules strongly coupled to quantized light fields,^170^ and were probably the first who considered it for plasmonic nanoantennas as well. They showed that the formation of polaritons provides new minima in the potential energy surfaces in which the excited nuclear wavepackets can be trapped such that the photochemical reaction will be slowed down. When the coupling becomes even more, a barrier, much larger than the thermal energy, appears and the photoisomerization process becomes almost completely suppressed (Figure 11h). Moreover, this potential energy barrier becomes even higher with the increased number of molecules coupled to the light mode, thus giving rise to a “collective protection” effect. The collective nature of polaritons leads to the formation of a polaritonic “supermolecule” involving the degrees of freedom of all molecules and allowing to trigger a many-molecule reaction by just a single photon,^171^ which circumvents the second law of photochemistry (also known as the Stark–Einstein law). However, only in their later work, a more accurate model for plasmonic nanoantennas was built, which accounts for the plasmon-induced dissipative processes.^389^ The most complete to date theory of plexcitonic strong coupling, which nonperturbatively treats the molecular electronic density relaxation upon polariton formation together with plasmon-molecule correlation, has appeared only recently.^388^ It extends the QED-coupled-cluster method^390,391^ already applied to optical cavities, to realistic plasmonic nanocavities, thus providing the full quantum description of both molecule and plasmonic subsystems. Particularly, the modification of the ground-state electronic density caused by the molecule-plasmon interaction was demonstrated for the realistic molecule and plasmonic nanocavity (Figure 11i), which is beyond the standard Jaynes-Cummings picture.

In concluding this section, we bring the reader’s attention to several promising examples of future directions in the field of plexcitonic photophysics and photochemistry. A recent proposal^392^ and its experimental realizations^393,394^ of molecular frequency optomechanical upconversion in plasmonic nanocavities can be potentially improved by reaching the vibrational strong coupling regime in these systems. Furthermore, recent studies of strong coupling of coherent phonons to excitons in different semiconductors^395,396^ can be enriched by coupling to plasmonic systems. Additionally, the influence of strong coupling between a plasmonic resonator and organic molecules on hot carrier generation is an important task, as usually these two processes are considered as separate contributions, and often to demonstrate the role of strong coupling, researchers strive to minimize the hot carrier contribution. The first theoretical studies on this subject have just begun to appear.^397^ In connection with the development of quantum technologies, it could be interesting to observe the influence of the strong coupling regime on the photon entanglement in plasmonic lattices.^398^ Finally, let us note a strong-coupling-induced symmetry breaking which can boost nonlinear optical processes.^399,400^

### 4.3 Open Cavities and Self-Hybridized Polaritons

Among open non-Hermitian systems, self-hybridized cavities have recently attracted special attention. Thanks to the proper size and sufficiently high contrast of the refractive index with the environment, such systems enable the hybridization of their own material resonance(s) and volumetric optical modes, thus, combining resonator and emitter subsystems within the same object. In this case, self-hybridized or cavity-free polaritons arise with the Rabi splitting approaching (but not exceeding) the so-called bulk polariton splitting limit. The latter has been pioneered by Hopfield^69^ in 1958 who studied bulk polaritons in 3D continuous semiconductors (see also Mills et al.^401^). The bulk polariton splitting is a function of the material resonance only (in the Lorentzian description determined in terms of oscillator strength, plasma frequency, and background permittivity) and does not depend on the parameters of the cavity. When the resonant material is confined in space, the splitting and detuning start to depend on the geometrical parameters. The calculation of such systems requires QNMs solutions obtained from the poles of the EM scattering matrix in the complex frequency plane,^61^ as we discussed in Section 2.4.

**Figure 12:**
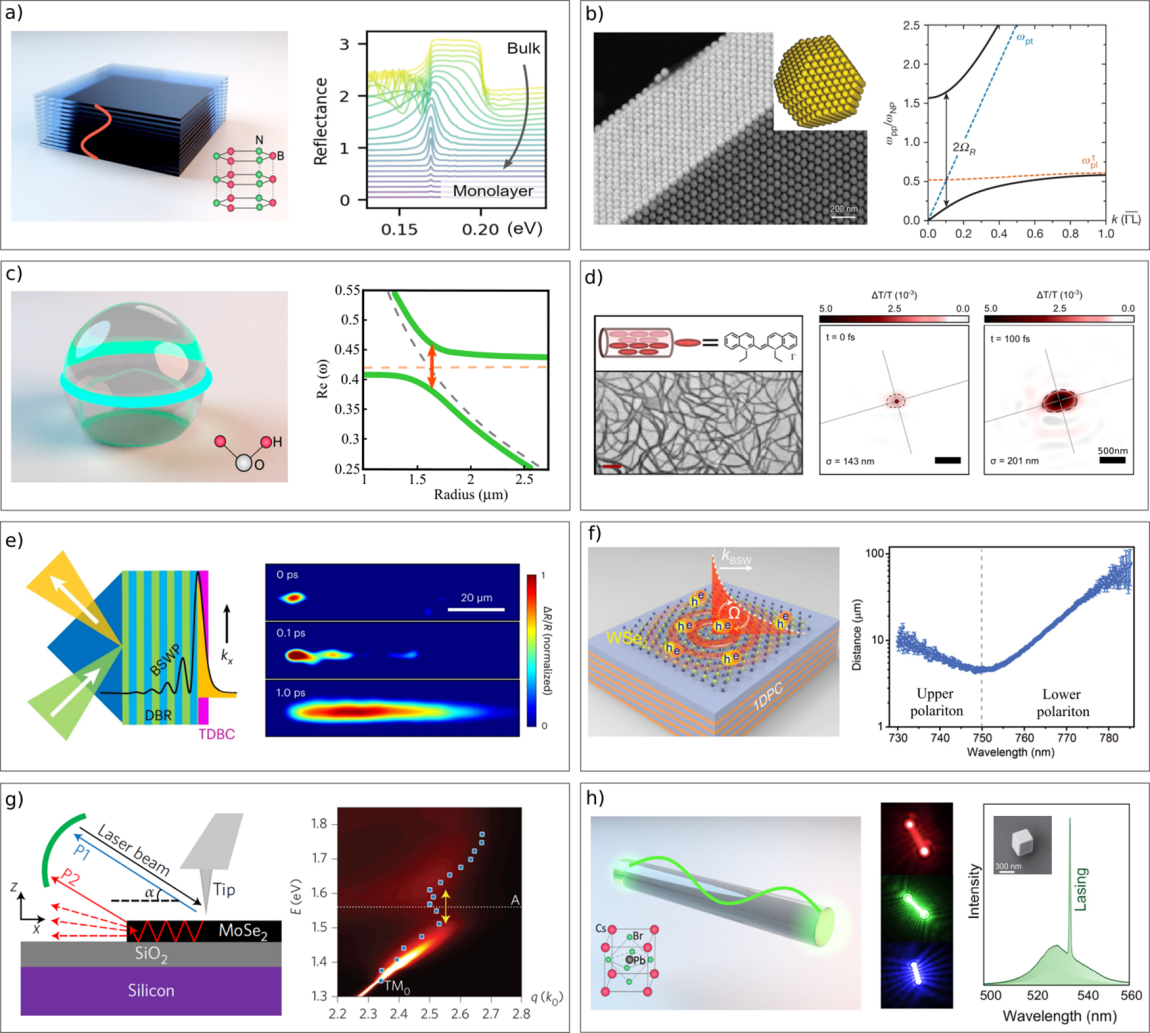
Self-hybridized polaritons in open cavities. (a) Schematic of an hBN slab with phononic self-polaritons (left) and reflectance spectra of an optically thick hBN on a polystyrene substrate. The traces for different thicknesses are offset for the sake of clarity (right). Reproduced from refs^61^^411^,. Copyright 2021 AIP Publishing and 2022 American Chemical Society. (b) Edge view and schematic of a face-centered-cubic crystal consisting of gold nanoparticles (left) and the plasmon-polariton dispersion of it with a large Rabi frequency (Ω_*R*_ = 1.4 ω_*pl*_) corresponding to the deep strong coupling regime (right). Reproduced from ref (414). Copyright 2020 Springer Nature. (c) Schematic of a water droplet with vibrational Mie self-polaritons (left) and the splitting of Mie TM-modes for a water sphere depending on its radius (right). Reproduced from ref (61). Copyright 2021 AIP Publishing. (d) Schematic and electron-microscopy images (scale bar 100 nm) of self-assembled nanotubular J-aggregates supporting self-hybridized exciton-polaritons (left) and femtosecond pump spot spatiotemporal dynamics showing the typical scale of enhanced exciton transport (right); here the dotted line shows the radial Gaussian standard deviation (σ). Reproduced from ref (60). Copyright 2021 Springer Nature. (e) Schematic of DBR structure coated with TDBC molecules supporting Bloch surface waves polaritons (left) and gradual expansion of the polariton cloud obtained with pump–probe reflection microscopy. Reproduced from ref (253). Copyright 2023 Springer Nature. (f) Schematic of Bloch surface wave exciton-polaritons in WSe_2_ slab on DBR substrate (left) and the polariton propagation length as a function of their wavelength (right). Reproduced from ref (422). Copyright 2022 the American Chemical Society. (g) Schematic of the experimental setup (left) and calculated dispersion color map with blue squares indicating experimental points (right) from the observation of self-hybridized exciton-polaritons in MoSe_2_ slabs beyond the light line. Reproduced from ref (424). Copyright 2017 Springer Nature. (h) Schematic of a perovskite CsPbBr_3_ nanowire with excitonic self-polaritons (left), optical images in the lasing mode operation of CH_3_NH_3_PbX_3_ [X = I (red), Br (green), Cl (blue)] perovskite nanowires (center), and emission spectra for a subwavelength CsPbBr_3_ nanocube (right). Reproduced from refs (61), (408), (425), and (426). Copyright 2021 AIP Publishing, 2018 the American Chemical Society, 2015 Springer Nature, and 2023 Song Jin.

Self-hybridized polaritons were first considered theoretically for spherical resonant materials.^402^ Around the same time, the first experimental observation of such polaritons was reported by Takazawa et al.^403^ using the exciton-polaritons in organic nanofibers. This topic was further developed by Liao et al.,^404^ who may have been the first to demonstrate room-temperature exciton-polariton lasing from self-assembled organic nanowires. However, in these works, the authors did not focus on the self-hybridized nature of the polaritons. Only in recent years have open systems attracted the attention of researchers, and many different implementations of self-hybridized polaritons have appeared within different geometries, material platforms, and frequency ranges. Among a large number of such works, we note realizations based on transition metal dichalcogenides,^405−407^ perovskites,^408,409^ and organic molecules^59,60,62,410^ with exciton-polaritons in the visible and near-infrared range, hexagonal boron nitride (hBN) with phonon-polaritons in the mid-IR range^411^ (Figure 12a), organic waveguide gratings with vibro-polaritons in the THz range,^412^ and even in plasmonic systems with plasmon–interband interactions in the UV range.^413^ Moreover, 3D crystals of plasmonic nanoparticles can reach the deep strong coupling regime (the coupling strength exceeds the plasmon frequency) when the plasmonic nanoparticles are ∼10-times larger than the interparticle gaps^414^ (Figure 12b).

One interesting example of self-hybridized polaritonic systems existing in nature is vibrational Mie self-polaritons in ∼1 μ m water droplets, which were recently shown theoretically to reach the ultrastrong coupling regime^61^ (Figure 12c). Interestingly, such droplet sizes are encountered in fogs, mists, and clouds and therefore could be present in atmospheric science measurements. Indeed, Arnott et al.^415^ have measured infrared spectra of laboratory clouds and observed some peculiarities in the 3000–3500 cm^–1^ range, although without explicitly interpreting these peculiarities in terms of self-hybridized Mie polaritons in water droplets. Nevertheless, such types of droplets could potentially find use as polaritonic platforms or as cavities for reaching strong coupling with various types of admixed molecules since higher-order whispering gallery modes possess exceptionally high Q-factors.

One may ask the question of whether strong coupling in open cavities could potentially be promising in terms of modification of material properties, such as polaritonic chemistry and exciton transport.^416^ In this regard, it has recently been proposed that polaritonic chemistry effects should be referenced with respect to bulk polaritons since such polaritons exist even in absence of any confinement.^417^ It has also been shown that open cavities (slabs) do not reach the same level of vibrational density of states as mirror-based Fabry–Perot microcavity polaritons do (but rather remain close to the level of bulk polaritons), suggesting that their effect on polaritonic chemistry would be minor.^417^ Thus, open cavities could potentially influence polaritonic chemistry only if they support high Q-factor modes.

In terms of exciton transport, open cavities could provide an additional benefit in comparison to ordinary mirror-based cavities. In particular, mirror-based Fabry–Perot cavities, due to the highly reflective external mirrors, achieve weakly broadened optical modes and therefore reach the strong coupling regime more easily than the cavity-free counterparts. However, the presence of external mirrors, in addition to complicating the fabrication, causes difficulties with charge injection and light outcoupling in prospective polaritonic devices. Open cavities with moderately confined photonic states enable the realization of strong coupling and, on the other hand, help overcome these obstacles. This makes them potentially interesting for future optoelectronic device applications. For instance, it has been predicted that exciton transport in organic materials can be enhanced when the molecules are strongly coupled to an electromagnetic mode.^418,419^ The delocalization of the exciton-polariton modes helps them bypass the disorder and other imperfections in organic semiconductors and thus overcome the exponential suppression of the transmission properties. Remarkably, this prediction was confirmed for organic layers not only in an artificial cavity with external mirrors^252^ or in plasmonic nanoparticle arrays,^254^ but even in bare nanocylinders of J-aggregates with self-hybridized polaritonic states^60^ (Figure 12d). In the latter experiment, when the refractive index of the organic material and the external optical environment were matched with that of oil, no ultrafast exciton-polariton transport was observed. That confirms the crucial role of polaritons in the exciton transport enhancement, as for their formation sufficiently large optical contrast is needed while oil can not affect the exciton transport. Furthermore, the excitonic components of exciton-polaritons can be manipulated by an external electric field, which due to strong exciton–photon coupling enables the realization of asymmetric light propagation in organic nanowire waveguides.^420^

Exciton transport enhancement in bare organic layers is interesting for device applications. In such systems, the polariton propagation length is typically on the order of hundreds of nanometers (Figure 12d). However, by coupling to Bloch surface waves supported by DBR substrates under the excitonic layer, the polariton propagation can be enhanced by up to 3 orders of magnitude both for disordered organic layers^253,421^ (Figure 12e) and crystalline semiconductors (Figure 12f).^422^ This is due to the high Q-factor, long lifetime, and large group velocity of the Bloch surface waves. A polaritonic mode with 80% photonic weight entirely overcomes the disorder and shows a ballistic propagation with distances ∼100 μ m and at a speed of two-thirds of the speed of light.

Furthermore, self-hybridized exciton–polaritons have been shown recently to relax the crystal symmetry selection rules that govern second-order nonlinearities.^423^ Usually, there is no second harmonic generation (SHG) from bulk crystals with inversion symmetry because the macroscopic nonlinear polarization vanishes for all even-order nonlinear processes. For example, a giant SHG signal from a MoS_2_ monolayer is almost completely suppressed in its bulk 2H-phase. However, self-hybridized polaritons can provide an asymmetric distribution of nonlinear polarization, thus giving rise to the resonant uncompensated bulk SHG signal. For the bulk WSe_2_ films of 140 nm thickness, the largest SHG enhancement (comparable to its monolayer SHG) was demonstrated for the DBR substrate combined with an air spacer.^423^

In addition to self-hybridized polaritons above the light line discussed in previous paragraphs, one can also realize strong coupling with guided modes beyond the light line (Figure 12g). This has been done with excitons in organic molecules^427^ and transition metal dichalcogenides,^424,428^ and phonons in hBN that were intermixed with vibrations of organic molecules.^429^ This type of polaritons will not be considered further; for additional references, we refer the reader to reviews by Basov et al.^430^ and Low et al.^431^ Finally, it is worth mentioning that guided modes in perovskite nanowires and nanoplates were also shown to self-hybridize strongly with excitons.^408,409^ Remarkably, by variation of the composition of perovskite nanowires, it is possible to tune the emission wavelength throughout the entire visible spectrum (Figure 12h). Furthermore, lasing, including that in the polaritonic regime, has been reported in such structures.^425,432,433^ A more complete list of observations of lasing and self-hybridized polaritons in different perovskite-based systems can be found in the reviews in references (434−436). The next section provides a detailed review of the polariton condensation effects and lasing in organic systems.

## 5 Condensation of Organic Exciton-Polaritons

Photons and excitons are bosons with an integer spin quantum number. They are ruled by Bose–Einstein statistics, which allow the occupation of the same quantum state by many bosons. This property is in contrast to the single state occupancy of Fermions, ruled by Fermi–Dirac statistics. From this important characteristic, it follows that, under certain conditions, it is possible that all bosonic particles occupy in equilibrium the lowest energy state of a system. This occupied ground state is known as a *Bose–Einstein condensate* (BEC). Following their observation in ultracold gases,^437,438^ Imamoğlu et al. proposed that a BEC could be realized with excitons in a semiconductor strongly coupled photons in an optical cavity.^439,440^ The orders of magnitude lower effective mass of exciton-polaritons compared to atoms, mainly due to the hybridization of excitons with photons, leads to a much higher condensation temperature (the critical temperature for BEC scales with the inverse of the effective mass of the bosons). The large binding energy of Frenkel excitons in organic semiconductors, larger than the thermal energy at room temperature, allows for condensation at room temperature, in contrast to the cryogenic temperatures needed for most inorganic semiconductors and atoms. This section focuses only on organic BECs. The interested reader on inorganic BECs is referred to the many excellent reviews in this topic.^441,442^

Exciton-polariton BECs produce a laser-like emission. Indeed, the light emitted from a macroscopically occupied quantum state is coherent. In this context, a photon laser and a BEC of exciton-polaritons show similar characteristics: a nonlinear regime beyond a threshold, temporal and spatial coherence, and spontaneous polarization build-up. Both phenomena require a cavity to provide coherence and, more importantly, bosonic stimulation. Bosonic stimulation, also known as Bose enhancement, is a process that occurs because of the occupation of the same quantum state. For Fermions, this occupation is not allowed, and the resulting effect is known as the Pauli blockade. Both Bose enhancement and Pauli blockade are a result of the symmetric and antisymmetric properties of the wave functions of bosons and Fermions, respectively. Focusing on bosons, the symmetric wave function leads to a transition probability of (1 + N) into a state where there are already N bosons. Hence, the name bosonic stimulation is the reason behind the phenomena of stimulated emission and scattering. To amplify this stimulation, the cavity plays the key role of preserving coherence so that the stimulation is enhanced.

**Figure 13:**
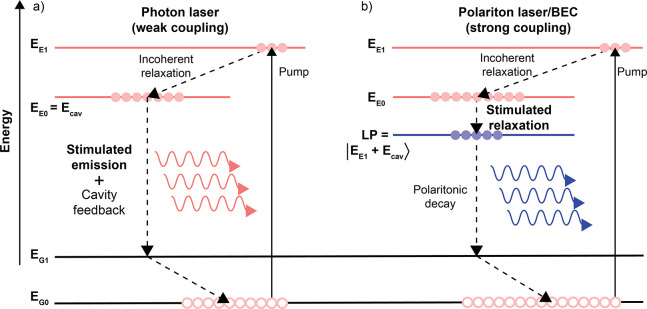
Schematic representation of the energy levels and relaxation processes in (a) a photon laser and (b) a polariton laser.

A major difference between photon lasing and condensation of exciton-polaritons lies in which species are coherent and, therefore, the stimulated process that becomes amplified. Figures 13a and b schematically show the steps involved in a photon laser and in the condensation of organic exciton-polaritons, respectively. The electronic ground and excited states of the molecule are *E*_*g*_ and *E*_*e*_, respectively, with several vibronic sublevels. Nonresonant pumping at an energy level, *E*_*E*1_, well above the excited states involved in the lasing/condensation is assumed. Following the pumping, there is a fast incoherent relaxation toward the lowest vibronic sublevel, *E*_*E*0_. This relaxation is nonradiative and mediated by molecular vibrations, leaving the 4-level system typically discussed in photon lasers.^443^ Excitons accumulated in the vibronic sublevel create a reservoir. In the photon laser schematics displayed in Figure 13a, the cavity is at the same energy level as the lowest vibronic sublevel. Coherence is provided by the cavity, which confines light into only a single optical mode. Excitons and photons are weakly coupled in a photon laser, which means that the coherence of the photons is not shared with the excitons. As a result, only photons emitted in the cavity mode preserve coherence, which amplifies the process of stimulated emission and leads to lasing. Population inversion comes as a result of the gain and loss balance: while gain is provided by amplification, loss is a consequence of absorption. The photon lasing threshold is achieved when both are in balance.

On the other hand, in the strong coupling regime (Figure 13b), the hybridization between excitons and cavity photons creates new eigenstates, the upper and lower polaritons. The lower polariton (LP) is at a lower energy than the vibronic sublevel *E*_*E*0_ of the uncoupled molecules, which defines the exciton reservoir and enables the relaxation from this reservoir to the LP. Coherence is now shared by the excitons that are strongly coupled to the cavity photons. This coherence is again possible thanks to the cavity, and is extended to excitons thanks to the strong coupling. Because the coherence is shared by excitons, the relaxation transition into the LP branch becomes a process that can be stimulated. The threshold for condensation depends on the critical density upon which all exciton-polaritons relax into the lowest energy state of the LP branch. Contrary to a photon laser, this threshold does not involve a balance of gain versus losses. Hence, population inversion is not required for polariton lasing.^440^ This lack of population inversion does not mean that losses do not matter. The buildup of the critical density takes time as excitons need to relax from the reservoir into the LP branch. However, the finite lifetime of polaritons decreases their density and demands a higher threshold to achieve condensation. The finite lifetime depends on both the radiative decay, as well as the nonradiative channels by which a polariton can decay. These decays make condensation of exciton-polaritons a competition between the relaxation rate that builds up the density and the losses that reduce it.

The origin of the emission in a condensate of exciton-polaritons is the finite lifetime of the LP. Since the emission originates from a single quantum state that is macroscopically occupied by bosons, it presents the same characteristics as a laser. However, the lifetime of exciton-polaritons is usually comparable or shorter than the thermalization rate, which means that exciton-polaritons form nonequilibrium condensates.^440,444^ Some researchers argue that reaching thermodynamic equilibrium is a necessary requirement to form a Bose–Einstein condensate,^444,445^ which is characterized by a population distribution that follows the expected Bose–Einstein distribution. While there is not yet consensus on the terminology that should be used when referring to polaritonic systems, the term “*polariton lasers*” usually refers to nonequilibrium exciton-polariton BECs.^446^

### 5.1 Evidence for Condensation of Exciton-Polaritons

**Figure 14:**
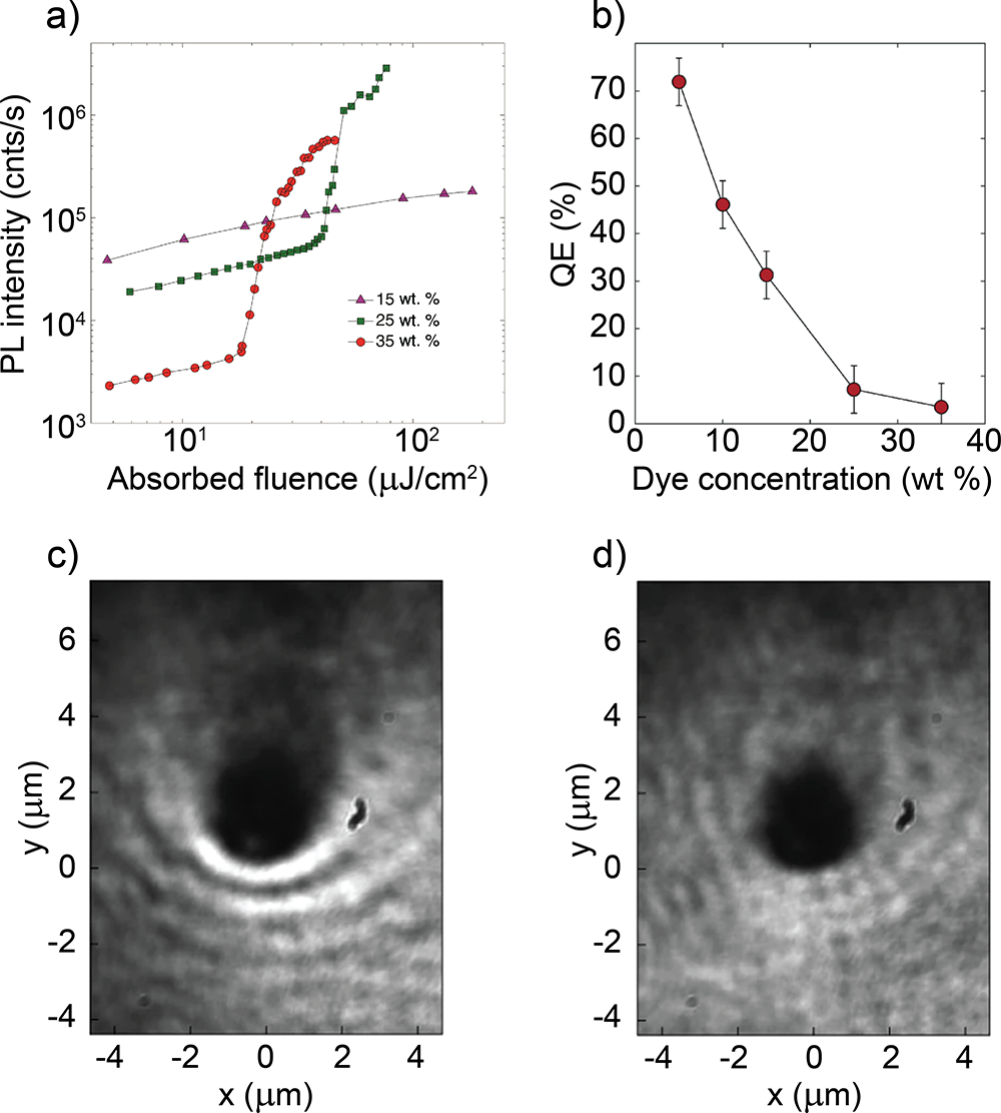
(a) Threshold curves of polariton lasers for different concentrations of organic molecules. (b) Emission quantum efficiency of the same molecules as a function of the concentration in the polymer matrix (PMMA). Reproduced from ref (18). Copyright 2017 Optica Publishing. (c) Scattering of an organic exciton-polariton fluid by a defect. (d) Superfluidic organic BEC moves friction-less along the defect. Reproduced from ref (447). Copyright 2017 Springer Nature.

The main differences between a photon laser and a polariton laser can be observed in the measurements of the emission above threshold. One of the most clear evidence of condensation is presented in Figure 14a and shows that the threshold is reduced when increasing the concentration of dye molecules in a polymer layer strongly coupled to surface lattice resonances in an array of metallic nanoparticles.^18^ Increasing the molecular concentration reduces the emission quantum yield of the dye due to concentration quenching, as shown in Figure 14b. In a photon laser, it is expected that increasing concentration will reduce the gain, which in turn would also increase the threshold, i.e. the opposite behavior than the one reported in ref (18). On the other hand, the increased density of molecules leads to an increased collective coupling strength. Also, the increased density of excitons at large dye concentrations results in a faster rate for stimulated scattering of exciton polaritons into the lower polariton states and the concomitant reduction of the condensation threshold.^106,376^

Another evidence of a polariton BEC, although more difficult to observe, is the formation of quantized vortices when the polariton condensate exhibits superfluidity.^447^Figure 14c shows that in the conventional fluid regime, an exciton-polariton fluid is scattered by a defect, which results in the formation of the fringes around the defect. Upon the transition to the superfluidic regime, the condensate moves friction-less and there is no scattering by the defect, which can be seen in the lack of fringes in Figure 14d.

It is common to also report a power-dependent blueshift in the energy of the polariton lasing as an indication for condensation.^448−450^ Photons do not interact with each other but excitons do. In inorganic condensates, this blue shift is a well-known effect due to the repulsive Coulomb interaction between the excitonic parts of the polaritons. In organics, however, exciton interactions are weak due to the localized nature of Frenkel excitons. Nevertheless, a blueshift is still observed upon increasing the excitation power above the condensation threshold. For a long time, it has not been clear what processes are responsible for this blueshift. The origin could lie in Frenkel exciton interactions,^448,450^ or it could be caused by the interplay of saturation effects and intermolecular energy migration.^451,452^ Rodriguez et al. suggested that as the pump fluence is increased, and thus the number of excitons injected, the saturation leads to a decrease of the Rabi splitting, which therefore blue shifts the lower polariton.^453^ The theoretical treatment by Arnardottir et al. using beyond-mean-field strong coupling quantum theory shows that the blue-shift originates from saturation effects, and can be described as an effective interaction between the polaritons.^451^

The extended spatial coherence of polariton lasers, beyond the excitation spot, can be used as another piece of evidence for condensation.^450,454,455^ This extended coherence is attributed to the enhanced exciton-polariton transport, as discussed in Section 4.3. Also, the decay of correlations above the condensation threshold has been shown to be nonexponential, in contrast to lasing phases.^456^

### 5.2 Formation of an Organic Polariton Condensate

Frenkel excitons are localized in a few molecules, which, as mentioned above, reduce exciton–exciton scattering and limit the relaxation to direct transitions from the reservoir to the bottom of the LP where condensation occurs. In Section 3.1.1, it was described that this kind of relaxation can be divided into a nonradiative channel, known as vibrationally assisted scattering (VAS), and a radiative channel, known as radiative pumping. VAS involves the emission of a molecular vibration, which allows the exciton to reduce its energy and transfer it to the LP branch. As described by Litinskaya et al.^106^ and Mazza et al.,^376^ the formation of a condensate imposes the condition that the molecular vibration needs to match exactly the energy difference between the reservoir and the bottom of the LP branch, i.e. *E*_*vib*_ = *E*_*Exc*_ – *E*_*LP*_ at (*k*_∥_ = 0). Most demonstrations of condensation with organic exciton-polaritons are based on VAS of the reservoir excitons,^15,16,18,448,457^ including the first observation by Kena-Cohen and Forrest.^15^ Because VAS is fixed to a certain vibrational level, the condensate is formed at a fixed energy, given by the molecular structure. Changing the parameters of the cavity, such as the Fabry–Perot thickness, does not shift the energy of the condensate. Ramezani et al. showed that the periodicity in arrays of silver nanoparticles defining open cavities does not have an influence on the condensation energy, even if the LP strongly depends on this period.^458^

Radiative pumping, on the other hand, was believed to have a smaller contribution to condensation. Although this mechanism was already proposed by Litinskaya et al.,^106^ early works on organic condensates did not observe clear signs of a direct radiative pathway assisting the formation of the condensate. However, several works in recent years have shown the presence of the radiative channel in the relaxation of excitons to the LP branch.^140,459−461^ Radiative pumping does not necessarily need to come from the same molecular species, but from any species with an emission spectrum overlapping with the LP branch.^140^ Akselrod et al. demonstrated that polariton lasing is possible using one specie in high concentration to form a lower polariton but with a slow relaxation rate, and using another specie in low concentration but high quantum yield to populate this lower polariton.^459^

Despite recent evidence, radiative pumping is not yet well understood. According to Mazza et al., it depends on the vibronic replica in the emission spectrum.^376^ The work of Groenhof et al. suggests that whether a molecule will exhibit a radiative decay rate into the lower polariton or not, depends on the Stokes’ shift of the emission.^118^ Grant et al. proposed that to facilitate the formation of polariton condensates, it is important that the molecule has a high quantum efficiency and a fast radiative rate.^460^ As mentioned by Mazza et al.^376^ and Groenhof et al.,^118^ radiative pumping can be the dominant relaxation channel in the formation of an organic condensate. It is therefore important to understand better this mechanism since it could lead to a reduction in the thresholds for condensation.

### 5.3 Prospects of BECs as Low Threshold Sources of Coherent Radiation

The generation of laser-like emission without population inversion makes low-threshold coherent emission one of the most promising applications of exciton-polariton BECs. Other applications of organic BECs exploiting the large nonlinearities include polariton transistors,,^462^ and quantum information processing and simulation.^463−466^ BEC emission or polariton lasing has been demonstrated in several organic systems, from single crystals to dye molecules and biomolecules. An advantage of organic systems over inorganic systems is that the samples can be solution processed by spin coating thin films.

**Figure 15:**
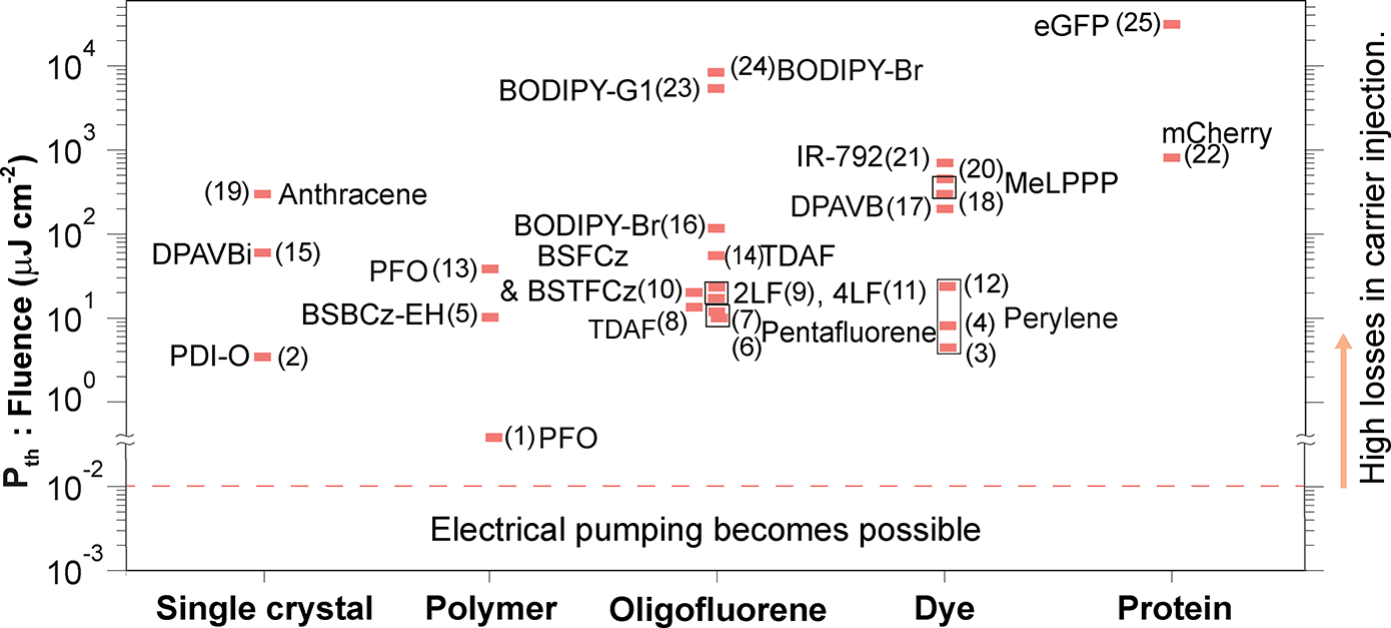
Polariton laser threshold in units of fluence (μJ cm^–2^) for several organic materials. The data are sorted by the type of organic material and also by increasing fluence. Note that no distinction is made between incident and absorbed fluence, as these two quantities are used in the literature. Single crystal: (1)-,^481^ (2)-,^457^ (15)-,^469^ (19)-;^15^ Polymer: (5)-,^471^ (13)-;^470^ Oligofluorenes: (6)-,^482^ (7)-,^128^ (8)-,^483^ (9)-,^476^ (10)-,^475^ and (11)-,^476^ (14)-,^448^ (16)-,^472^ (23)-,^163^ (24)-;^484^ Dyes: (3)-,^485^ (4)-,^486^ (12)-,^18^ (17)-,^478^ (18)-,^487^ (20)-,^16^ (21)-;^477^ Proteins: (22)-,^480^ (25)-.^479^ The red dashed line indicates the value of the threshold below which electric pumping would be able to overcome the losses associated with carrier injection. The black squares around some threshold values group the same organic material with the intention to distinguish it from other compounds in situations where they are very close together in threshold value.

The most important milestone in polariton lasing will arguably be the demonstration of condensation with electrically injected carriers. Thresholds reported for optically pumped condensation in organic systems are still too high, and a remaining open question is whether electrically driven condensation can be achieved.^467^ These thresholds are on the order of μJ cm^–2^ to mJ cm^–2^. Figure 15 shows a nonexhaustive collection of works reporting threshold values for organic polariton lasers after the first observation by Kena-Cohen and Forrest in 2010 using a single crystal of anthracence.^15^ Since then, condensation has been observed in many other organic systems, including other single crystals,^15,457,468,469^ polymers,^72,449,470,471^ oligofluorenes,^128,163,448,472−476^ dyes,^16,18,458,477,478^ proteins,^479,480^ and hybrid systems.^242^ The value of the threshold for condensation has been reduced by more than 2 orders of magnitude, with the lowest threshold reported in the order of just a few μJ cm^–2^. Figure 15 also shows the estimated value in terms of absorbed fluence from which electrical pumping of polariton lasing may become possible. This estimation is made by considering that state-of-the-art VCSELs have thresholds on the order of ∼10 A cm^–2^. A transformation of Amperes into electrons and electrons into photons gives an initial estimation of 15–30 W cm^–2^. Considering an average regenerative amplifier with a repetition rate of 1–5 kHz and a pulse width of 100–150 fs and multiplying this width by the power obtained before we find a value of 2–5 nJ cm^–2^ for electrical pumping.

Consequently, the greatest challenge of organic polariton lasers is reduction of the threshold. To understand how this threshold of condensation can be reduced, it is worth modeling the formation of the condensate. Various models can be used to describe polariton lasing and condensation. A comprehensive overview of these models can be found in ref (467). The strong similarities between photon lasers and condensates of exciton-polaritons can be used to model the dynamics of the condensate by a master equation, as introduced by Mazza et al.:^376^
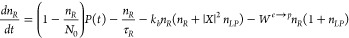
28a
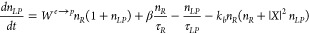
28bwhere *n*_*R*_ and *n*_*LP*_ are the densities of excitons in the reservoir and of exciton-polaritons in the LP branch, respectively, with lifetimes τ_*R*_ and τ_*LP*_. There is an exciton–exciton annihilation term with a rate *k*_*b*_. The absorbed power from pump *P*(*t*) is a time-dependent Gaussian function for a pulsed laser. Absorption occurs at a much faster rate than relaxation and can be considered to be instantaneous in this model. The term *N*_0_ represents the total molecular concentration and takes into account the saturation effects. The β-term corresponds to the fraction of spontaneous emission that contributes to the population of the LP, similar to the case for conventional lasers. This term represents a spontaneous process, and is thus incoherent. Therefore, it is impossible to have condensation based only on this contribution. The relaxation rate from the exciton reservoir into polaritons is given by *W*^*e*→*p*^ and the term (1 + *n*_*LP*_) introduces the bosonic final state stimulation. *W*^*e*→*p*^ is a sum of all the transitions into the bottom of the LP branch and therefore includes VAS and radiative pumping.

Condensation is the result of the accumulation of exciton-polaritons in the bottom of the LP branch. The threshold corresponds to the amount of excitons that needs to be injected to create a sufficiently high density of polaritons that is needed to form the condensate. Looking at the temporal dynamics of the polariton density *dn*_*LP*_/*dt*, we can see that the terms *W*^*e*→*p*^ and β contribute to the accumulation of polaritons, while the terms τ_*LP*_ and *k*_*b*_ reduce their density. Of particular importance are *W*^*e*→*p*^ and τ_*LP*_, i.e., the relaxation rate and the polariton lifetime, respectively. These two rates point out two pathways where efforts must concentrate to reduce the threshold of condensation. On the one hand, the relaxation rate depends on molecular properties, such as the concentration, the vibrational spectrum, and the density of states of these vibrations, as well as the emission spectrum and the emission quantum yield of the molecule as a function of the concentration. Improving these properties would lead to a faster relaxation rate. On the other hand, the polariton lifetime depends on the lifetime of the cavity mode, which can be tuned by controlling the quality of the mirrors or the scattering of radiation to far-field. Improving the cavity will increase the polariton lifetime and reduce the condensation threshold.

## 6 Summary and Future Directions

The purpose of this review was to highlight the formation of the research field now commonly referred to as polaritonic chemistry or polaritonic photochemistry/photophysics. In 2012, Wineland and Haroch were awarded the Noble prize in physics for their work in the 1980s regarding the manipulation of individual quantum systems.^488^ In essence, they placed individual atoms or ions (both can be regarded as close to perfect two-level systems) inside state-of-the-art optical cavities. Thus, creating a system with so minute energy losses that the very small light–matter coupling achieved was larger than energy dissipation from the system (the definition for entering the strong coupling regime). About 20 years later, organic dyes were shown to be able to enter the strong coupling regime.^11,13^ This was perhaps a priori not obvious because, on the one hand, organic dyes show very large interactions with light, but on the other hand, they also show very broad optical transitions. However, by collectively coupling organic dyes to a single cavity mode, we reached the strong coupling regime at room temperature. The third step worth to highlight is the realization that organic molecules are more than a two-level system, which was done about ten years ago.^19^ At this point, a molecule able to perform an isomerization reaction on the excited state surface was shown to change the rate of this reaction in the strong coupling regime. Chemistry was thus shown to be affected, marking the starting point of the research field of polaritonic chemistry.

We have discussed two methods of providing the electromagnetic mode to which molecular transitions can be coupled: the Fabry–Perot and plasmonic resonators. Both of these provide enough electric field strength to enter the strong coupling regime, but they have some differences that are worth highlighting. The Fabry–Perot cavity is a well-defined system with clear boundaries, and the electromagnetic mode volume is approximately equal to the physical cavity volume. Further, all molecules can be placed in, for instance, nodes or antinenes inside the cavity. However, it is common that the entire cavity volume is filled with dyes, and the macroscopically observed collective coupling is then dependent on the summation of interactions inside the cavity. Plasmonic cavities do not have such clear boundaries as the Fabry–Perot cavity. The electric field strength decreases with distance from the particles, but there is no physical barrier that defines the cavity. However, the enhancement of the electromagnetic field can be much larger because the mode volume is smaller. The consequence is that although approximately the same collective coupling strengths can be reached with both types of cavities, the number of molecules that are coupled to each cavity mode is smaller for a plasmonic cavity than for a Fabry–Perot cavity. The plasmonic cavity even enables strong coupling at the limit of a few or even single molecules. The coupling number is predicted to be of importance to transitions toward polaritonic states. Comparing photochemical reactions or transitions in Fabry–Perot and plasmonic cavities could therefore form a viable route to experimentally target this important number.

It is interesting to linger on size scales because they govern possible implementations. To couple a transition in the visible regime of the electromagnetic spectrum, a Fabry–Perot cavity with a thickness of about 100–150 nm can provide a suitable optical mode. Thus, there is no insurmountable barrier when it comes to size scales to implement strongly coupled systems into organic electronics, although this requires the use of highly absorbing dyes that are present at high concentrations. In fact, it is more than likely that unintended strong coupling in organic electronic devices has already been performed since the refractive index mismatch is enough to drive some systems into the strong coupling regime.^59−62^ There are some attractive features of polaritons that can boost organic electronic devices. The most noticeable of these features are the negligible reorganization energy, which can reduce voltage losses, their delocalized nature, which can provide long-range energy transfer, and the narrow emission lines.

The compatibility between strong coupling and organic electronics puts the prospect of polaritonic Bose–Einstein condensates into perspective. The ability of organic materials that are collectively coupled to a cavity mode to reach the BEC threshold, even at room temperature, is fascinating. Furthermore, when comparing the current state-of-the-art BEC thresholds with possible electron injection rates, this research direction gets a clear momentum where basic science becomes technologically relevant. Closing the orders of magnitude difference of threshold in state of the art condensates to reach technological relevance can be approached by a combination of cavity optimization, increasing the fluence of electrical pumping, and optimizing the relaxation paths of excitons to the LP branch.

With regard to recent developments in fabrication methods for Fabry–Perot microcavities, it is worth mentioning an intriguing platform based on Casimir self-assembly, which promises to become a possible future direction for polaritonics. This approach relies on harnessing the quantum electrodynamics of Casimir-Lifshitz forces to assemble microcavities in a liquid environment. It has first been suggested theoretically^489−491^ and later realized experimentally using a delicate balance between attractive and repulsive Casimir-Lifshitz^492^ forces in a system comprising a few micron gold nanoflake in ethanol solution trapped above an interface consisting of a continuous gold mirror and a Teflon spacer.^493^ Later, a similar approach was used to create self-assembled Fabry–Perot microcavities in an aqueous solution, where the equilibrium distance (∼100–200 nm) between the gold nanoflakes, which were playing the role of microscopic mirrors in the cavity, was controlled by balancing the electrostatic repulsion and Casimir-Lifshitz attraction.^494^ Furthermore, excitons in a few-layer WSe_2_ flake positioned between the mirrors were used to reach the strong coupling regime with the optical mode of the self-assembled microcavity. Such an arrangement allows to dynamically modulate the microcavity resonance by radiation pressure, salinity of the solution, and temperature and in doing so tune the system in and out of the strong coupling regime.^494^ The latter may be advantageous for a direct comparison between the weak and strong coupling regimes in one and the same material system, without the need of preparing several samples and thus potentially suffering from experimental inhomogeneity. This platform could be further enriched by introducing the critical Casimir forces into the self-assembly process, which, as was demonstrated recently, can even counteract Casimir-Lifshitz attraction.^495^

Optimal dyes for strong exciton–photon coupling should absorb as much as possible and emit at unity quantum efficiencies after being processed into the relevant high-concentration physical state. Although new highly absorbing dyes with high quantum yields of emission have been constantly developed, these are not always suitable for strong coupling experiments. This is because of the requirement of processing in as high a concentration as possible. Typically, when organic dyes are used, these are blended into a polymer matrix in order to prevent dye–dye interactions that significantly perturb their photophysical properties. Further, organic dyes are typical planar structures with a high tendency to form crystallites with different orientations within an otherwise amorphous material. To enable clear spectroscopic signatures, a well-defined spectral material is needed. To achieve this material, single dye or composite crystals can be used,^496,497^ with a completely amorphous material being the alternative. To achieve an amorphous material, the affinity of crystallization needs to be reduced at the same time as the glass fragility is reduced, which can be achieved by taking advantage of entropy.^498−500^ Thus, there is still a large optimization potential when it comes to dye development within this research field.

Considering the progress already made since the birth of the research field of polaritonic chemistry, it is not unreasonable to see considerable landmarks before the end of this decade. These could be the development of dyes that are optimized for high-concentration conditions. Such materials would increase the coupling strengths and emission efficiencies. Thus, reducing threshold values for BEC, together with technology development on the electron injection side, would allow for electrically pumped BECs. Further help would come from tools that allow for direct measurements of the effective delocalization number in energetically disordered systems (i.e., organic dyes in condensed phase) to enable more accurate predictions between cavity parameters and transfer rates, which will be essential for building polaritonic enhanced organic electronics. Future directions should also focus on situations when the formation of polaritons enables processes to proceed at efficiencies beyond those achievable when using traditional chemistry and physics. This rather shows that polaritons can provide small changes in the chemistry and physics. Finally, strong exciton–photon coupling enables the creation of hybrid light–matter states with unique properties; one just needs to use those properties to one’s advantage.
